# Structural Design Strategies of Polymer Matrix Composites for Electromagnetic Interference Shielding: A Review

**DOI:** 10.1007/s40820-021-00707-2

**Published:** 2021-08-18

**Authors:** Chaobo Liang, Zhoujie Gu, Yali Zhang, Zhonglei Ma, Hua Qiu, Junwei Gu

**Affiliations:** 1 Key Laboratory of Functional Nanocomposites of Shanxi Province, College of Materials Science and Engineering, North University of China, Taiyuan, 030051 China; 2 Shaanxi Key Laboratory of Macromolecular Science and Technology, School of Chemistry and Chemical Engineering, Northwestern Polytechnical University, Xian, 710072 China; 3 Research and Development Center, Guangdong Suqun New Materials Co., Ltd, Dongguan, 523000 China

**Keywords:** Polymer matrix composites, Electromagnetic interference shielding, Structural design

## Abstract

With the widespread application of electronic communication technology, the resulting electromagnetic radiation pollution has been significantly increased. Metal matrix electromagnetic interference (EMI) shielding materials have disadvantages such as high density, easy corrosion, difficult processing and high price, etc. Polymer matrix EMI shielding composites possess light weight, corrosion resistance and easy processing. However, the current polymer matrix composites present relatively low electrical conductivity and poor EMI shielding performance. This review firstly discusses the key concept, loss mechanism and test method of EMI shielding. Then the current development status of EMI shielding materials is summarized, and the research progress of polymer matrix EMI shielding composites with different structures is illustrated, especially for their preparation methods and evaluation. Finally, the corresponding key scientific and technical problems are proposed, and their development trend is also prospected.

## Highlights

- The review discusses the key concepts, loss mechanisms and test methods of electromagnetic interference (EMI) shielding.
- The research progress of polymer matrix EMI shielding composites with different structures is detailedly illustrated, especially their preparation methods and corresponding evaluations.
- The key scientific and technical problems for polymer matrix EMI shielding composites with different structures are proposed, and their development trend are prospected.

## Introduction

With the rapid development of 5G technology, artificial intelligence, Internet of Things, big data and their wide applications in unmanned systems, high-speed communications, industrial internet, future energy, aerospace and other fields, the development level of information technology has become a reflection of the comprehensive national power. However, the electronic devices can generate undesirable electromagnetic interference (EMI) to the outside world during operation, which not only affects the normal operation of nearby electronic devices, but also increases the risk of related workers suffering from headaches, depression, immune deficiency, and other diseases [1–3]. Therefore, there is an urgent need for high-efficiency EMI shielding materials to attenuate electromagnetic waves to protect the normal operation of electronic equipment and human health. In this context, devoting to the development of various high-performance EMI shielding materials has become a research hotspot [4–6].

Polymer matrix composites have been widely used in the field of EMI shielding due to their low density, corrosion resistance, competitive prices and good processability [7, 8]. However, most of the polymer matrix is inherently insulating, which severely limits the applications in electronic products, new energy vehicles, medical equipments, flexible circuit boards and other fields with high EMI shielding requirements [9, 10]. Currently, researchers have effectively solved the problem of poor EMI shielding performance of polymer matrix composites by compounding conductive fillers with resin matrix. Meanwhile, carbon nanotubes, graphene, metal nanowires/particles, and MXene have been widely used as conductive fillers for polymer matrix composites, and the current status of polymer matrix EMI shielding composites based on different types of conductive fillers has also been outlined and discussed in detail [11–13].

However, the current polymer matrix composites have disadvantages such as low electrical conductivity and poor EMI shielding properties compared to the metallic materials. How to improve the EMI shielding performance of polymer matrix composites through efficient structural design has become a technical difficulty and scientific problem that needs to be solved urgently [14]. In addition to the significant influences of the amount and type of conductive fillers on the EMI shielding performance of polymer matrix composites, the structural design of composites and the distribution orientation of the conductive fillers in the resin matrix are equally important. So far, few reviews based on different structural types of polymer matrix EMI shielding composites have been reported. Therefore, a comprehensive overview of the relationship between structure and EMI shielding performance of polymer matrix composites can help identify possible research directions to overcome the bottleneck of the existing technology and promote the further development of polymer matrix composites in the field of EMI shielding.

In view of this, the review first discusses the key concepts, loss mechanisms and test methods of EMI shielding. Then, the current development status of EMI shielding materials is totally summarized, and the research progress of polymer matrix EMI shielding composites with different structures is detailedly illustrated, especially their preparation methods and corresponding evaluations. Finally, the key scientific and technical problems for polymer matrix EMI shielding composites with different structures are proposed, and their development trend is prospected, which is expected to provide some guidance for the design, developments and industrial applications of the high-performance polymer matrix EMI shielding composites.

## Overview of EMI Shielding

At present, the most effective means to eliminate electromagnetic radiation pollution is the implementation of EMI shielding. EMI shielding means that to cut off or attenuate the electromagnetic waves generated by the work of high-frequency circuits to the outside world. This not only eliminates interference to neighboring electronic equipment and radiation to the human body, but also ensures that electronic equipment itself is not affected by external equipment [15, 16]. Due to the rapid development of electrical equipment such as satellite communications, radio and television, people have been unable to avoid exposure to a wide range of electromagnetic radiation pollution in most parts of the world [17–19].

### EMI Shielding Classification

In daily life, the high-precision electronic components and human being are often exposed to different kinds of electromagnetic radiation threats. It is crucial to use effective protection measures to shield electromagnetic radiation. According to the electromagnetic wave interference sources, EMI shielding can be classified as electrostatic field shielding, magnetic field shielding and electromagnetic field shielding.

#### Electrostatic Field Shielding

**Fig. 1:**
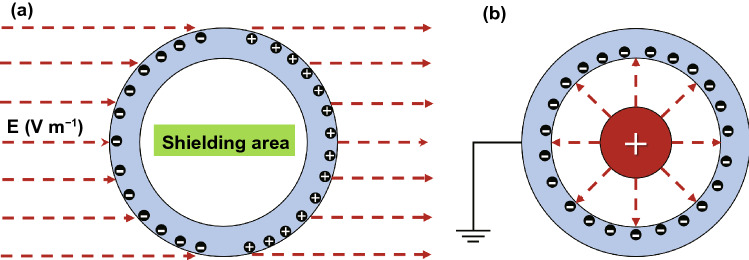
Schematic diagram of EMI shielding mechanism of **a** external electric field and **b** internal electric field

For the electrostatic field radiation, a conductive cavity can be applied to protect the electromagnetic sensitive equipment (external electric field shielding), as shown in Fig. 1a. Under the effect of an external electrostatic field, the left and right sides of the conductive cavity induce the opposite electric charges with equal intensity. According to the principle of electrostatic balance, the conductive cavity is an equipotential body with the internal potential difference and electric field intensity of zero. Therefore, the electromagnetic sensitive equipment inside the conductive cavity is protected from the interference of the external electrostatic field. In addition, for the electrostatic field radiation, the radiation source can also be shielded by the conductive cavity to protect the electromagnetic sensitive equipment (internal electric field shielding), as shown in Fig. 1b. Under the effect of the charged body inside the conductive cavity (generating electrostatic field), the inner surface of the cavity induces an equal negative charge opposite to the charged body, while the outer surface of the cavity induces an equal positive charge. As the outer surface is connected to the earth through the ground wire, the positive charge induced on the outer surface and the electric field outside the conductive cavity disappear, thereby realizing the protection of electromagnetic sensitive equipment outside the conductive cavity [5].

#### Magnetic Field Shielding

**Fig. 2:**
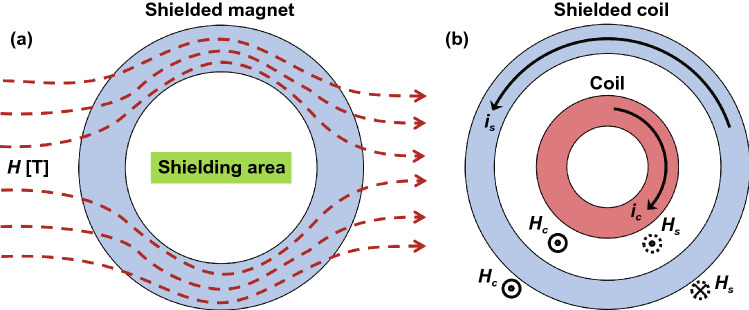
Schematic diagram of EMI shielding mechanism of **a** low-frequency magnetic field and **b** high-frequency magnetic field

For the shielding of low-frequency magnetic field, the shielding cavity with low reluctance is used in parallel with the protected space area, and the low-frequency magnetic field always tends to pass through the shielding cavity preferentially to realize the shielding protection of the internal space in the cavity, as shown in Fig. 2a. The higher permeability results in the lower magnetic resistance of the materials. Therefore, iron, cobalt, nickel and their alloys with high magnetic permeability are ideal materials for low-frequency magnetic field shielding. For the shielding of high-frequency magnetic field, the shielding cavity is used to generate an induced current under the external magnetic field, which excites an induced magnetic field opposite to the original magnetic field. As shown in Fig. 2b, the two magnetic fields achieve the shielding of high-frequency magnetic field by canceling each other. The high-frequency current in the coil generates a high-frequency radiating magnetic field, and the induced current in the shielding coil is opposite to that in the coil according to Faraday's law of electromagnetic induction. Therefore, the magnetic fields generated by the two coils are always in opposite directions outside the shielded coil, and the shielding of high-frequency magnetic field is achieved by canceling each other [5].

#### Electromagnetic Field Shielding

In practical applications, most shielding refers to electromagnetic field shielding. In the alternating electromagnetic field, the shielding of electric and magnetic fields must be considered together as the electric field and magnetic field always exist simultaneously. The electromagnetic radiation can be divided into near-field and far-field radiation depending on the electromagnetic waveform. When the distance between the electromagnetic radiation source and the shielding body is less than /2 ( is the wavelength of the electromagnetic radiation source), it is defined as the near-field radiation. There is a phase difference of 90° between the electric and magnetic fields of electromagnetic waves in the near-field radiation. The energy of the electric and magnetic fields of electromagnetic waves attenuates as the distance between the electromagnetic radiation source and the shield increases. If the electromagnetic radiation source has a large current with low voltage, the electromagnetic radiation source is dominated by a magnetic field. At this time, only the magnetic field shielding should be considered, and the electric field shielding should be ignored. If the electromagnetic radiation source has a small current with high voltage, the electromagnetic radiation source is dominated by an electric field. At this time, only the electric field shielding can be considered, and the magnetic field shielding can be ignored. When the distance between the electromagnetic radiation source and the shielding body is larger than /2, it is defined as the far-field radiation. The electric and the magnetic fields of the electromagnetic waves in the far-field radiation have the same phase and are perpendicular to each other. Therefore, the electric and magnetic fields of the electromagnetic waves have the same energy, and their shielding cannot be ignored. According to Maxwell's classical electromagnetic theory, the electric and magnetic fields of high-frequency electromagnetic waves are always closely linked together to form a unified electromagnetic field rather than exist in isolation. Therefore, as long as the radiation from the electric or magnetic field is completely shielded, the other radiation will no longer exist in practical applications.

### Mechanism of EMI Shielding

**Fig. 3:**
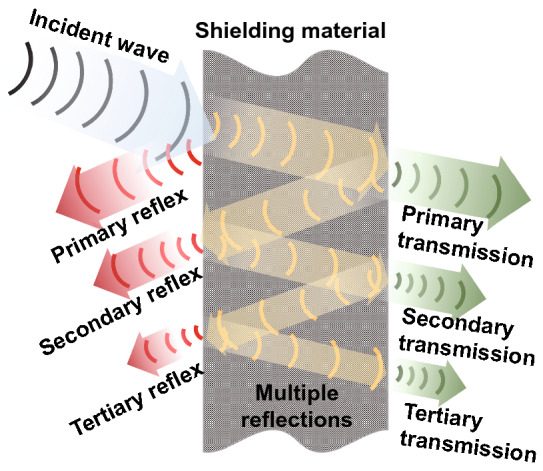
Schematic diagram of EMI shielding mechanism based on transmission line theory

In order to understand the EMI shielding more intuitively, various shielding mechanisms have been proposed, such as the eddying effect theory, electromagnetic field theory, and transmission line theory. Among these, the transmission line theory has been most widely recognized [20–22]. The transmission line theory means that the electromagnetic waves passing through the shielding materials will be affected by three different shielding mechanisms, including the reflection, absorption and multiple reflection. Specifically, when the electromagnetic waves are incident on the surface of the shielding materials, the electromagnetic waves are reflected due to the impedance mismatch between the shielding materials and the external free space. The unreflected electromagnetic waves enter the inside of the shielding materials and are continuously attenuated by dielectric loss and magnetic loss. In addition, the electromagnetic waves can be dissipated through multiple reflections at the interface inside the materials. The residual electromagnetic waves will pass through the shielding materials and become transmitted waves [11, 23]. The EMI shielding mechanism is shown in Fig. 3.

The attenuation or loss of electromagnetic waves is one of the key indicators for the materials to evaluate the ability of shielding electromagnetic waves. It can qualitatively describe the difference in electromagnetic wave intensity before and after the shielding process. EMI shielding effectiveness (EMI SE, unit: dB) can be used to quantitatively evaluate the ability of materials to shield electromagnetic waves. It is defined as the ratio of the electric field strength (*E*), magnetic field strength (*H*) or power (*P*) of the electromagnetic waves before entering the shielding materials and those after passing through the shielding materials, expressed as Eq. 1 [7, 24]:

1 $$ {\text{SE}} = 20\lg \left( {\frac{{E_{t} }}{{E_{i} }}} \right) = 20\lg \left( {\frac{{H_{t} }}{{H_{i} }}} \right) = 20\lg \left( {\frac{{P_{t} }}{{P_{i} }}} \right) $$

In Eq. 1, the subscripts *i* and *t* represent the incident electromagnetic waves and transmitted electromagnetic waves, respectively. *E*_*i*_, *H*_*i*_, and *P*_*i*_ represent the incident electric field, magnetic field and power intensity respectively, while *E*_*t*_, *H*_*t*_, and *P*_*t*_ represent the transmitted electric field, magnetic field, and power intensity, respectively. According to Schelkunoff theory, EMI SE is defined as the sum of three shielding mechanisms including reflection (SE_*R*_), absorption (SE_*A*_) and multiple reflections (SE_*M*_) for attenuation of electromagnetic waves by shielding materials.

2 $$ {\text{SE}}_{T} = {\text{SE}}_{R} + {\text{SE}}_{A} + {\text{SE}}_{M} $$

In Eq. 2, SE_*R*_, SE_*A*_ and SE_*M*_ can be calculated by Eqs. 3–5:

3 $$ {\text{SE}}_{R} = 168.2 + 10\lg \left( {\frac{{\sigma_{r} }}{{f \mu_{r} }}} \right) $$

4 $$ {\text{SE}}_{A} = 131.43t\sqrt {f \mu_{r} \sigma_{r} } $$

5 $$ {\text{SE}}_{M} = 20\lg \left( {1 - e^{{\frac{ - 2t}{\delta }}} } \right) = 20\lg \left( {1 - 10^{{\frac{{{\text{SE}}_{A} }}{10}}} } \right) $$

where, *σ*_*r*_ is the relative electrical conductivity, *μ*_*r*_ is the relative magnetic permeability, *f* is the frequency of electromagnetic waves (Hz), *t* is the thickness of the shielding materials (m), *δ* is the skin depth (m), and *r* is the distance from the radiation source to the shielding materials (m). From Eq. 5, SE_*M*_ and SE_*A*_ are correlated with each other. SE_*M*_ plays a crucial role in the geometric morphological structures. However, SE_*M*_ can be neglected for relatively thick shielding materials, as the amplitude of electromagnetic waves is negligible when they reach the second boundary of the shielding materials. In other words, when the SE_*A*_ of the shielding materials is ≥10 dB, the SE_*M*_ can be neglected [12]. It can also be seen from the equation that SE_*R*_ is inversely related to *f* and *μ*_*r*_, and positively related to *σ*_*r*_. SE_*A*_ is positively related to the *t*, *f*, *μ*_*r*_, and *σ*_*r*_ of the shielding materials. Therefore, the increase of magnetic properties can improve the SE_*A*_ and reduce the SE_*R*_ of the shielding materials. The increase of electrical conductivity can simultaneously improve SE_*R*_ and SE_*A*_ of the shielding materials, thereby enhancing their EMI shielding performances [25, 26].

### Test Methods for EMI Shielding

**Fig. 4:**
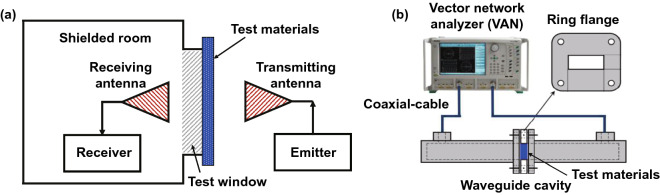
Schematic diagram of the test principle of **a** shielded room method and **b** network analyzer method

For the tests of EMI SE in the near-field and far-field radiation, the researchers developed the corresponding shielded room method and the network analyzer method, as shown in Fig. 4a, b respectively. The shielded room method is a common technique used for the near-field EMI SE measurement. The test process is as follows: A specific test window is opened on the wall of a shielded room made of metal plates, and electromagnetic wave transmitters and electromagnetic wave receivers are placed on opposite sides of the test window, respectively. The signal intensity of the electromagnetic waves with the test window free of the materials (electric field strength *E*_0_, magnetic field strength *H*_0_ or power *P*_0_) and the signal intensity of the electromagnetic waves with the test window covered by the materials (electric field strength *E*_1_, magnetic field strength *H*_1_ or power *P*_1_) are both tested. Thereby, the ratio of the two kind of received signal intensity is used as the EMI SE of the test materials, as shown in Eq. 6 [2]:

6 $$ {\text{SE}} = 20\lg \left( {\frac{{E_{0} }}{{E_{1} }}} \right) = 20\lg \left( {\frac{{H_{0} }}{{H_{1} }}} \right) = 20\lg \left( {\frac{{P_{0} }}{{P_{1} }}} \right) $$

The network analyzer method is a common technique used for the far-field EMI SE measurement. The EMI SE in the far field can be calculated by the measured *S*-parameters (*S*_11_, *S*_21_, *S*_22_, *S*_12_) from a vector network analyzer, where *S*_*ij*_ indicates the transmission from port *j* to port *i*. Based on the *S*-parameters, the reflectance (*R*), absorbance (*A*) and transmittance (*T*) can be calculated by Eqs. 7, 8, and 9, respectively [3]:

7 $$ R = \left| {S_{11} } \right|^{2} = \left| {S_{22} } \right|^{2} $$

8 $$ T = \left| {S_{12} } \right|^{2} = \left| {S_{21} } \right|^{2} $$

9 $$ A = 1 - R - T $$

SE_*T*_, SE_*R*_ and SE_*A*_ can be obtained by calculating *R*, *A* and *T* as shown in Eqs. 10, 11, and 12, respectively:

10 $$ {\text{SE}}_{T} = 10\lg \left( \frac{1}{T} \right) = 10\lg \left( {\frac{1}{{\left| {S_{12} } \right|^{2} }}} \right) $$

11 $$ {\text{SE}}_{R} = 10\lg \left( {\frac{1}{1 - R}} \right) = 10\lg \left( {\frac{1}{{1 - \left| {S_{11} } \right|^{2} }}} \right) $$

12 $$ {\text{SE}}_{A} = 10\lg \left( {\frac{1 - R}{T}} \right) = 10\lg \left( {\frac{{1 - \left| {S_{11} } \right|^{2} }}{{\left| {S_{12} } \right|^{2} }}} \right) $$

## Classification of EMI Shielding Materials

The shielding technology plays a vital role in controlling or reducing the electromagnetic radiation pollution, among which high-performance EMI shielding materials are the key to the realization of shielding technology [27, 28]. According to the EMI SE values, EMI shielding materials can be divided into various categories: (a) low-level shielding materials (10–30 dB), which can be used in low-end shielding applications; (b) medium-level shielding materials (30–60 dB), which can meet most industrial-grade shielding needs; (c) high-level shielding materials (60–90 dB), which can meet the shielding needs of the military industry and aerospace fields; (d) high-precision shielding materials (>90 dB), which can meet the shielding needs of high-precision and high-sensitive precision electronic devices [11, 29]. According to the matrix, EMI shielding materials can be divided into metallic EMI shielding materials, magnetic EMI shielding materials and conductive polymer EMI shielding materials. In order to obtain a more comprehensive understanding of EMI shielding materials, this section provides a brief classification and overview of current EMI shielding materials.

### Metallic EMI Shielding Materials

Conventional metallic materials (e.g., metal sheets, metal blocks, and metal foams, etc.) have been widely used in the field of EMI shielding due to their high electrical conductivity [30–32]. Park et al. [33] prepared the NiFe/Cu multilayer sheets by a DC magnetron sputtering technology. The results show that the 4 μm thick NiFe/Cu multilayer sheets have a batter EMI SE of 90 dB compared with the single-component Cu or NiFe sheets in the frequency range of 0.7–10 GHz, owing to the multiple reflections of electromagnetic waves between the interfaces of NiFe/Cu multilayer sheets. Sambyal et al. [34] synthesized the FeSiAl sendust/metal hybrid materials with magnetic cores and highly conductive metal shells by electroless plating Ag or Ni metal on the surface of FeSiAl sendust. The results show that the FeSiAl sendust coated with Ag and Cu exhibits an absorption-dominated EMI SE of 65 and 58 dB, respectively, in the frequency range of 300 kHz–10 GHz. Song et al. [35] used the coaxial cable method to fabricate the EMI SE of AZ31 sheets (Mg-3 wt% Al-1 wt% Zn) with different texture strengths. The results show that the EMI SE of the AZ31 sheets reaches 72–98 dB in the frequency band of 30–1500 MHz. Although the metal materials exhibit ultra-high EMI shielding performance, their shallow skin depth leads to the high reflectivity of electromagnetic waves, causing the secondary radiation pollution in the environment. In addition, the metal materials have disadvantages such as high density, expensive, easy to corrode and difficult to process, which greatly limit their wider applications. Moreover, the metal materials usually show magnetic and wave leakages at the joints, which will seriously affect their EMI SE [36–38].

### Magnetic EMI Shielding Materials

According to the classical electromagnetic theory, the magnetic materials can shield the electromagnetic waves mainly through the hysteresis loss, eddy current loss, domain wall resonance, ferromagnetic resonance, and natural resonance mechanisms [39, 40]. Common magnetic shielding materials are mainly divided into two categories: the metal alloy materials based on iron, cobalt, nickel, etc., and the metal oxide materials such as carbonyl iron, carbonyl nickel, ferrite, and garnet [41, 42]. Fei et al. [43] prepared the zeolitic imidazolate framework-67/cellulose nanofiber (ZIF-67/CNF) aerogel via the directional freeze-casting technique, and then obtained magnetic Co/C@CNF aerogel by the high-temperature thermal reduction. The results show that the EMI SE of the magnetic Co/C@CNF aerogel after thermal reduction at 900 °C reaches 35 dB. Ren et al. [44] prepared the graphene nanosheets/carbonyl iron-nickel alloy/cyanate ester (GNSs/CINAP/CE) composites by the combination of solution blending and hot pressing. The results show that the EMI SE of the GNSs/CINAP/CE composite reaches 55 dB when the contents of CINAP and GNSs are 20 wt% and 5 wt%, respectively. Luo et al. [45] used the electroless plating and spraying techniques to coat silver (Ag) nanoparticles, ferroferric oxide (Fe_3_O_4_) nanoparticles and polydimethylsiloxane (PDMS) on the surface of polypropylene (PP) fabrics. The EMI SE of the obtained PP@Ag@Fe_3_O_4_@PDMS fabric reaches 56 dB. The magnetic metal materials generally have high magnetic permeability and strong saturation magnetization, and are suitable for the radiation shielding in the field of high-frequency weak electric field or low-frequency magnetic field. In addition, among many loss mechanisms, only natural resonance loss can dissipate electromagnetic waves in the GHz band. Thus, the magnetic materials have some limitations in practical applications, so they are usually used in combination with conductive materials [46–48].

### Conductive Polymer EMI Shielding Materials

Compared with the traditional metal materials, the conductive polymer materials developed in recent years have become more promising alternatives for EMI shielding due to their high specific strength, corrosion resistance, low cost, and easy processing [49–51]. Depending on the conductive mechanism, the conductive polymer materials used for EMI shielding can be divided into two categories: One is the intrinsic conductive polymer materials, and the other is the composite conductive polymer materials [52–54].

#### Intrinsic Conductive Polymer Materials

The matrix polymer of intrinsic conductive polymer materials possesses conductive ability, such as polypyrrole (PPy), polyaniline (PANI) and polythiophene (POT) [55]. Qiu et al. [56] prepared PANI doped with hydrochloric acid (PANI-HCl), PANI doped with camphorsulfonic acid (PANI-CSA), and PANI doped with phosphoric acid (PANI-H_3_PO_4_). It is noted that the PANI-CSA exhibited the highest *σ* and EMI SE of 1.28 S cm^−1^ and 21 dB, respectively, owing to the more complete oxidation, higher crystallinity and larger crystal size. Muller et al. [57] prepared poly-3, 4-ethylenedioxythiophene/bacterial cellulose nanofiber composite films by in-situ chemical oxidation method, with the *σ* up to 1.5 S cm^−1^. Currently, the conductivity of intrinsic conductive polymer materials is relatively low, and the preparation process is complicated. The process often requires the introduction of some high-cost and corrosive dopants through chemical methods. Moreover, the modified conductive polymer materials generally have the disadvantages of poor stability, high rigidity and difficulty in melt, thus leading to the difficulties in the later processing and molding processes [58–60].

#### Filled Conductive Polymer Composites

The filled conductive polymer composites refer to multi-phase composite system with conductive/EMI shielding functions prepared by compounding the polymer matrix with conductive fillers. The filled conductive polymer composites can be used as polymer matrix EMI shielding composites. The commonly used polymer matrixes include polyvinyl alcohol (PVA), polyethylene (PE), polylactic acid (PLA), polyurethane (PU), etc. [61–63]. The conductive fillers are mainly divided into three categories: carbon-based fillers such as graphite, reduced graphene oxide (rGO), multiwalled carbon nanotubes (MWCNTs), carbon black (CB), metal-based fillers such as silver nanowires (AgNWs), copper nanosheets (CuNSs), aluminum powder, composite fillers such as transition metal carbon/nitride grafted with ferric oxide (MXene@Fe_2_O_3_), graphene grafted with ferroferric oxide (rGO@Fe_3_O_4_), silver-plated carbon fiber [64–66]. Liang et al. [67] prepared the graphene/epoxy composites by doping functionalized graphene into epoxy resin matrix by a solution blending method. The results show that the graphene/epoxy composite shows a *σ* of 0.1 S cm^−1^ and an EMI SE of 21 dB when the content of graphene is 15 wt%. Li et al. [68] embedded a copper-plated graphene fiber mesh into the PDMS film, and the results showed that the *σ* and EMI SE of PDMS film reached 3.21 × 10^5^ S m^−1^ and 74 dB, respectively. Gu et al. [69] prepared the silver nanowire (AgNW)/CNF composite film by the combination of vacuum-assisted filtration and hot pressing. The results show that the AgNW/CNF composite film shows a high *σ* of 5.57 × 10^5^ S m^−1^ and an excellent EMI SE of 101 dB when the content of AgNWs is 50 wt%. Compared with the intrinsic conductive polymer materials, the composite conductive polymer materials (or polymer matrix EMI shielding composites) have been widely used in many fields such as aerospace, energy transportation, sporting goods, construction facilities, and defense industry thanks to their high strength, easy processing, good flexibility and low production cost, etc. [70–72].

## Research Progress of Polymer Matrix EMI Shielding Composites with Different Structural Designs

The preparation methods have great effects on the distribution of fillers and the microstructures, thereby influencing the overall performance of polymer matrix EMI shielding composites. Table 1 summarizes the EMI shielding performance of polymer matrix composites with different structural types. It can be seen that the structure, density and thickness play a decisive role in the EMI shielding performance of the composites. Therefore, it is crucial to use a suitable preparation method to fabricate the polymer matrix EMI shielding composites that can meet the practical application requirements. Depending on the different structural designs, polymer matrix EMI shielding composites can be classified into homogeneous structure, segregated structure, porous structure, layered structure and preformed structure.

**Table 1:** Comparison of EMI shielding performances of the polymer matrix composites with different structures

Type	Materials	Thickness (mm)	EMI SE (dB)	Frequency (GHz)	Density (g cm^−3^)	SSE (dB cm^3^ g^−1^)	SE/t (dB cm^−1^)	SSE/t (dB cm^2^ g^−1^)	Refs
Homogeneous polymer composites	GnPs@PDA-MWCNTs/PPSU	3	62	8–12	/	/	207	/	[75]
	nAg-MWCNTs/NBR	0.02	45	0.030–1.5	/	/	225	/	[76]
	ABS/CB	1.1	21	8.2–12.4	/	/	190	/	[77]
	ABS/CF	1.1	35	8.2–12.4	/	/	318	/	[77]
	ABS/MWCNTs	1.1	51	8.2–12.4	/	/	464	/	[77]
	MWCNTs/PVDF	2	56	8–12	0.79	71	280	354	[80]
	SCF/EVA	3.5	34	8–12	/	/	97	/	[81]
	PP/EPDM/NCGF	2	41	8.2–12.4	/	/	205	/	[82]
	PPy/PDA/AgNW	/	48	8–12	0.28	171	/	611	[85]
	PANI-MWCNTs	2	39	12.4–18.0	/	/	195	/	[86]
Segregated polymer composites	graphite/PE	1	26	26–37.5	/	/	260	/	[94]
	CB/UHMWPE	2.1	33	8.2–12.4	/	/	157	/	[95]
	rGO/PS	2.5	45	8.2–12.4	/	/	180	/	[96]
	MXene@PS	2	62	8.2–12.4	/	/	310	/	[97]
Porous polymer composites	f-G/PVDF	/	20	8–12	/	/	/	/	[105]
	PMMA/GNPs-MWCNTs	2.5	36	8–12	0.6	60	144	240	[106]
	AgNS/epoxy	2	42	8–12	0.172	244	210	1221	[107]
	MWCNTs/WPU	2.3	50	8.2–12.4	0.126	397	217	1722	[109]
	RGO/LDC	2	49	8.2–12.4	0.008	6125	245	30,625	[110]
	AgNW/CNF	2	40	8.2–12.4	0.0017	23,888	200	178,235	[111]
	MXene/CNF	2	35	8.2–12.4	0.0015	23,633	175	118,167	[112]
	AgNW/PVB/MS	5	60	8–12	0.0019	31,579	120	63,158	[116]
	Fe_3_O_4_@MXene/GF/PDMS	1	80	8.2–12.4	/	/	800	/	[117]
	PU@PDA@Ag	5	84	8.2–12.4	0.032	2625	168	5250	[118]
Layered polymer composites	ANF-MXene/AgNW	0.091	80	8.2–12.4	1.63	8769	8791	5379	[124]
	CNF@MXene	0.035	40	8.2–12.4	1.62	25	11,429	7029	[125]
	MXene/c-PANI	0.04	36	8.2–12.4	/	/	9000	/	[126]
	Fe_3_O_4_@rGO/MWCNTs/WPU	0.8	35.9	8.2–12.4	/	/	449	/	[40]
	PVA/MXene	0.027	44.4	8.2–12.4	1.76	25	16,444	9343	[129]
	Ag/NWF/FeCo@rGO/WPU	0.4	77	2–18	/	/	1925	/	[130]
	Fe_3_O_4_@rGO/T-ZnO/Ag/WPU	0.5	87	8.2–12.4	/	/	1740	/	[131]
	Cu-Ag/ITO/PET	0.05	26	8–40	/	/	5200	/	[137]
	AgNW/PVA/PET	/	44	8.2–12.4	/	/	/	/	[138]
	AgNW/PDDA	/	31	8–12	/	/	/	/	[139]
	AgNW/MXene/PET	/	49	8.2–12.4	/	/	/	/	[140]
Preformed polymer composites	Graphene/PDMS	5	42	8.2–12.4	/	/	84	/	[146]
	GF/h-Fe_3_O_4_/PDMS	2	70	8.2–12.4	1.179	59	350	297	[147]
	Epoxy/wood-derived carbon	2	27.8	8–12	1.17	24	139	119	[148]
	Fe_3_O_4_/TAGA/epoxy	3	35	8.2–12.4	/	/	117	/	[149]
	TCTA/epoxy	2	74	8.2–12.4	1.2	62	370	308	[150]

### Homogeneous Polymer Matrix EMI Shielding Composites

The homogeneous polymer matrix EMI shielding composites are prepared by the uniform compounding of polymer matrix and conductive fillers through solution blending, melt blending and in-situ polymerization methods. The homogeneous polymer matrix EMI shielding composites have been widely used in practical industrial production due to their simple preparation process [73, 74].

#### Solution Blending Method

**Fig. 5:**
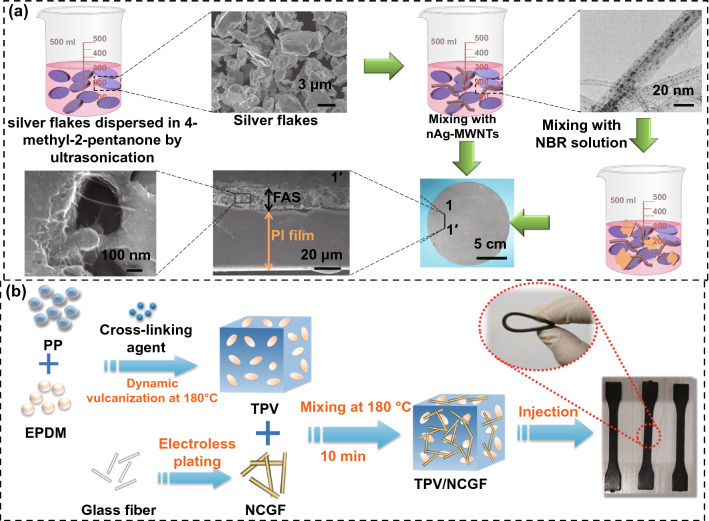
Schematic illustrating the fabrication of **a** nAg-MWNTs/NBR and **b** PP/EPDM/NCGF composites [76, 82]. Copyright © 2013 Elsevier

The solution blending method refers to fabricating the polymer matrix EMI shielding composites by dispersing the conductive fillers in the polymer matrix using a suitable solvent and then removing the solvent. In the solution mixing system with lower viscosity, the polymer and the conductive fillers can be uniformly mixed to ensure the formation of conductive pathways inside the final composites. He et al. [75] dissolved the polydopamine-modified graphite nanosheets (GnPs@PDA), MWCNTs and polyphenylsulfone (PPSU) in N, N-dimethylformamide (DMF), and then the GnPs@PDA-MWCNTs/PPSU composite powder was obtained by flocculation under an ice-water bath. Finally, the GnPs@PDA-MWCNTs/PPSU composites were obtained after hot pressing. The results show that the EMI SE of the composites reaches 62 dB when the content of GnPs@PDA-MWCNTs is 17.6 vol%. Kwon et al. [76] first dissolved the nitrile butadiene rubber (NBR) and MWCNTs grafted with silver nanoparticles (nAg-MWCNTs) in a toluene solvent, and then completely removed the toluene solvent with continuous stirring. Finally, the nAg-MWCNTs/NBR composites were obtained after curing (Fig. 5a). The results show that the EMI SE of the nAg-MWCNTs/NBR composites reaches 45 dB when the content of nAg-MWCNTs is 83 wt%. Al-Saleh et al. [77] fabricated the ABS/CB, ABS/carbon nanofibers (CF), and ABS/MWCNTs composites via the similar method. When the contents of the three fillers is 15 wt%, the ABS/CB, ABS/CF and ABS/MWCNTs composites exhibit EMI SE values of 21, 35, and 51 dB, respectively. The solution blending method can significantly reduce the viscosity of the composite system, thereby improving the dispersion of the fillers in the polymer matrix. However, the method also has significant disadvantages such as the needs to add a large amount of solvent during the preparation process. Many polymer materials can only swell but not dissolve in the solvent, and the incomplete subsequent treatment of the solvent can shorten the service life of the materials. Moreover, the removal of the solvent will bring the environmental problem and cost concern. Therefore, the industrial large-scale production of the solution blending method still needs further exploration [78, 79].

#### Melt Blending Method

The melt blending method refers to the homogeneous mixing of the molten polymer and conductive fillers by a mixing equipment above the viscous flow temperature of the polymer matrix, after cooling, the polymer matrix EMI shielding composites are obtained. Compared with the solution blending method, the melt blending method has simple operation, low cost, and no third-party solvent. The method has a wide range of industrial applications, and most of the thermoplastic EMI shielding composites can be prepared by this method. Wang et al. [80] used MWCNTs as conductive filler and polyvinylidene fluoride (PVDF) as matrix to prepare the MWCNTs/PVDF composites through the melt blending and compression molding. The results show that the EMI SE of the MWCNTs/PVDF composites reaches 56 dB when the MWCNT content is 15 wt%. Das et al. [81] used short carbon fiber (SCF) as conductive filler, natural rubber (NR) and ethylene–vinyl acetate copolymer (EVA) as matrix, and fabricated the SCF/NR and SCF/EVA composites by the similar method. When the content of SCF is 17.5 wt%, the SCF/NR composites exhibit an EMI SE of 20 dB. However, the EMI SE of SCF/EVA composites reaches the same level with only 8 wt% of SCF owing to the low viscosity of EVA. Duan et al. [82] used nickel-coated glass fiber (NCGF) as the conductive filler, polypropylene (PP) and ethylene-propylene-diene monomer (EPDM) as the matrix to prepare the PP/EPDM/NCGF composites via the melt blending and injection molding (Fig. 5b). The EMI SE of the resultant composites reaches 41 dB when the NCGF content is 15 wt%. The melt blending method has been widely used in actual industrial production due to its good processing performance and low-cost advantage. However, the high shear forces generated by the equipment during the mixing process can damage the structure of the conductive fillers, thereby leading to the decrease of the EMI shielding performances. The polymer matrix composites prepared by the melt blending method require high filler content to achieve the construction of three-dimensional conductive network, so the selection of conductive fillers is limited [83, 84].

#### In-situ Polymerization Method

**Fig. 6:**
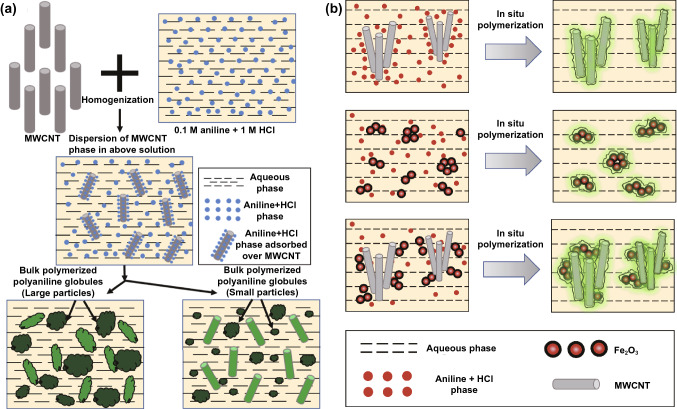
Schematic illustrating the fabrication of **a** PANI-MWCNTs and **b** PANI-MWCNTs/Fe_2_O_3_ composites [86, 87]. Copyright © 2008 Elsevier

The in-situ polymerization method refers to uniformly mixing the conductive fillers and the polymer monomers, and the polymerization reaction is initiated by adding an initiator to obtain the polymer matrix EMI shielding composites. This method have no very high shear force, so the surface structure of the fillers is not easy to be destroyed. The in-situ polymerization process can promote the uniform and stable dispersion of the conductive fillers, so it has more applications. Wang et al. [85] synthesized the PPy/PDA matrix by an in-situ polymerization and then obtained the PPy/PDA/AgNW composites by mixing with the AgNWs. The results show that when the AgNW content is 50 wt%, the *σ* and EMI SE of PPy/PDA/AgNW composites are 1206 S cm^−1^ and 48 dB respectively, which are 120,000 times and 6.9 times higher than those of pure PPy. Saini et al. [86] fabricated the PANI-MWCNTs composites by an in-situ polymerization based on free radical chemical oxidation (Fig. 6a). The results show that when the MWCNT content is 25 wt%, the *σ* and EMI SE of the composites are 19 S cm^−1^ and 39 dB, respectively, which are 9.5 times and 1.4 times higher than those of pure PANI. Yun et al. [87] introduced the iron trioxide (Fe_2_O_3_) nanoparticles into the PANI-MWCNTs composites prepared by in-situ polymerization, and further improved the EMI SE of composites (Fig. 6b). The above researches show that the composites prepared by the in-situ polymerization method exhibit a strong interface interaction between the conductive fillers and polymer matrix, and the conductive fillers have good dispersibility in the polymer matrix. However, the conductive fillers usually require surface modification, which will damage the physical and chemical properties of the fillers [88–90].

### Segregated Polymer Matrix EMI Shielding Composites

**Fig. 7:**
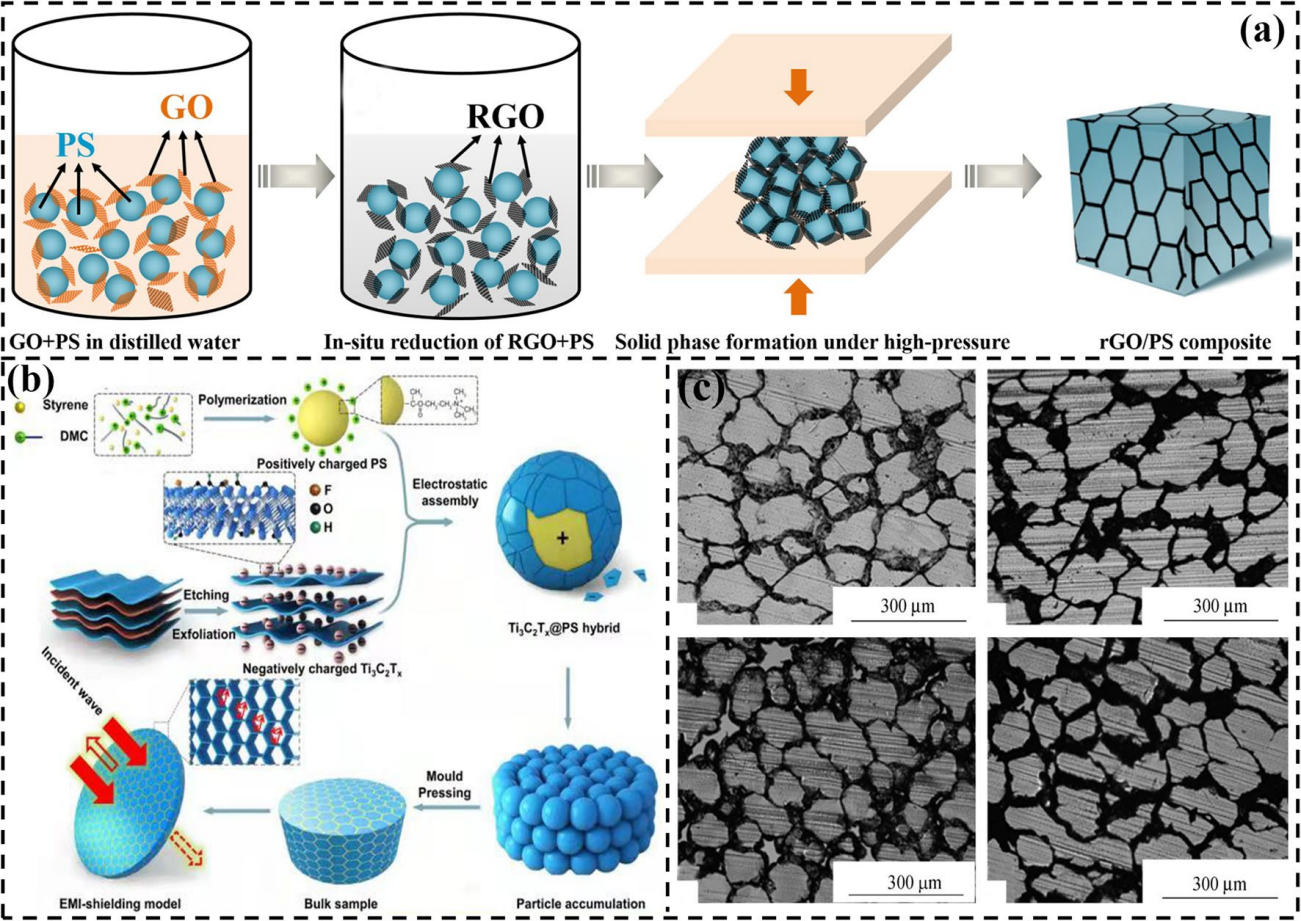
Schematic illustrating the fabrication of **a** rGO/PS and **b** MXene@PS composites [96, 97]. **c** SEM images of CB/UHMWPE composites [95]. Copyright © 2014 Wiley–VCH. Copyright © 2017 Wiley–VCH

The segregated polymer matrix EMI shielding composites are prepared by hot pressing of polymer particles coated with conductive fillers on the surface at high temperature. For homogeneous polymer matrix EMI shielding composites, the conductive fillers are randomly dispersed in the polymer matrix. In contrast, the conductive fillers are selectively distributed between the micro-zone interfaces of polymer matrix in the segregated polymer matrix EMI shielding composites. The probability of interlap between the conductive fillers is significantly increased, which facilitates the formation of perfect conductive networks at a low content of conductive fillers. Currently, the conductive fillers used to construct the segregated polymer matrix EMI shielding composites are mainly divided into two categories: micro-scale conductive fillers (graphite and CB) and nano-scale conductive fillers (graphene, MWCNTs and MXene) [91–93]. Vovchenko et al. [94] prepared the graphite/PE composites with a segregated structure based on the PE particles coated with graphite by a thermo-compression molding. The results show that the *σ* and EMI SE of graphite/PE composites reach 1.23 S m^−1^ and 26 dB, respectively, when the graphite content is 5.0 vol%, which are 17.6 times and 2.6 times higher than those with a homogeneous structure, respectively. Cui et al. [95] prepared the CB/ultra-high molecular weight polyethylene (UHMWPE) composites via the hot pressing of UHMWPE particles coated with CB obtained by a high-speed ball milling. The results show that the CB is selectively distributed in the interfacial region of the UHMWPE matrix, forming a typical segregated structure (Fig. 7c). The *σ* and EMI SE of the CB/UHMWPE composites are 14.1 S m^−1^ and 33 dB, respectively, with the CB content of 15 wt%. Yan et al. [96] coated the rGO on the surface of polystyrene (PS) particles by the in-situ reduction, and then compressed the obtained composite particles into the segregated rGO/PS composites by a high-pressure solid-phase compression molding (Fig. 7a). The rGO is selectively distributed in the interface between PS particles, instead of uniformly distributed in the PS matrix. The rGO/PS composites with a segregated structure can effectively reduce the percolation threshold and significantly improve the electrical conductivity. The EMI SE of the segregated rGO/PS composites reaches 45 dB when the rGO content is 3.47 wt%. Sun et al. [97] loaded the negatively charged MXene onto the surface of positively charged PS microspheres by an electrostatic self-assembly, and then prepared the highly conductive segregated MXene@PS nanocomposites by pressing (Fig. 7b). The MXene forms the effective three-dimensional conductive networks in the PS matrix. The resultant MXene@PS nanocomposites not only exhibit a low percolation threshold of 0.26 vol%, but also have an excellent *σ* of 1081 S/m and an high EMI SE of 62 dB.

The above studies show that although the micro-scale conductive fillers such as graphite and CB are low-cost and not easily agglomerated in the polymer matrix, the *σ* and EMI SE of the prepared polymer matrix composites are still not satisfied even at a high content of conductive fillers. The nano-scale conductive fillers such as rGO and MXene can easily form the conductive pathways within the polymer matrix due to their large specific surface area and excellent conductive property, thereby improving the *σ* and EMI SE of the polymer matrix composites. However, the nano-scale conductive fillers are costly and highly prone to agglomeration in the polymer matrix. In addition, the conductive fillers form a single continuous network structure and the polymer particles tend to exist in isolation after the polymer particles covered with conductive fillers are hot pressed, resulting in poor mechanical properties of polymer matrix EMI shielding composites. Therefore, while maintaining the excellent EMI shielding performance of segregated polymer matrix EMI shielding composites, further optimization of mechanical properties and manufacturing costs is the key to achieving large-scale applications [98–100].

### Porous Polymer Matrix EMI Shielding Composites

The porous polymer matrix EMI shielding composites use polymer matrix as the support skeleton, which is then modified or loaded with conductive fillers. Compared with homogeneous and segregated polymer matrix EMI shielding composites, the porous polymer matrix EMI shielding composites have the advantages of low cost, good toughness, and low density. At the same time, the porous structure can contribute to the multiple reflection and absorption of electromagnetic waves, which will further improve the EMI shielding performance of the composites. Currently, the foaming method, sol–gel method and template method are widely used to prepare the porous polymer matrix EMI shielding composites [101–103].

#### Foaming Method

**Fig. 8:**
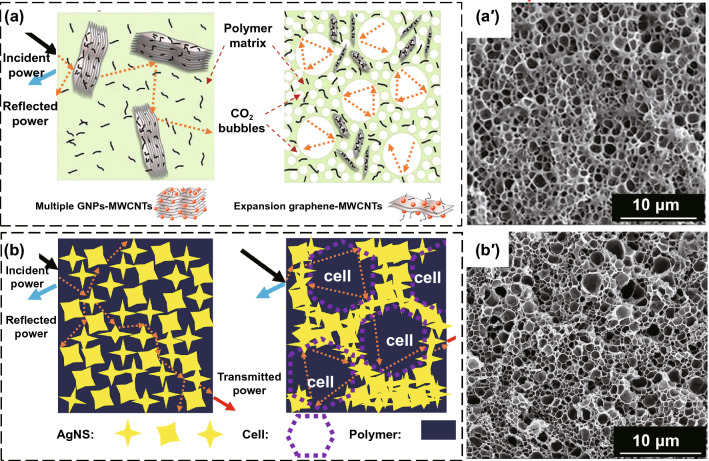
Schematic diagram and SEM images of **a** PMMA/GNPs-MWCNTs and **b** AgNS/epoxy composites with homogeneous and porous structures [106, 107]. Copyright © 2018 Elsevier. Copyright © 2019 Elsevier

The foaming process includes the chemical foaming method and physical foaming method. The chemical foaming method refers to mixing a foaming agent (azo compounds, sulfonyl hydrazide compounds and nitroso compounds, etc.) into the polymer matrix composites, and the foaming agent can decompose gas during the heating process to foam the composites. The physical foaming method means that the gas produced by the supercritical fluids (CO_2_, N_2_, butane, pentane, etc.) in a thermodynamically unstable state is nucleated, grown, and stabilized to achieve the foaming of polymer matrix EMI shielding composites [104]. Eswaraiah et al. [105] prepared the functionalized graphene (f-G)/PVDF composites with a porous structure by chemical foaming using functionalized graphene (f-G) as the conductive filler, PVDF as the polymer matrix, and azobisisobutyronitrile (AIBN) as the foaming agent. The results show that when the f-G content is 0.5 wt%, the EMI SE of the f-G/PVDF composites are 18 and 20 dB at the frequencies of 1–8 and 8–12 GHz, respectively. Zhang et al. [106] prepared the PMMA/GNPs-MWCNTs composites with porous structures by the direct blending and supercritical CO_2_ foaming techniques, using MWCNTs and graphene nanoplates (GNPs) as the conductive fillers and polymethyl methacrylate (PMMA) as the matrix. (Fig. 8a). The results show that when the contents of MWCNTs and GNPs are 4 wt% and 1.5 wt%, respectively, the EMI SE of the porous PMMA/GNPs-MWCNTs composites reaches 36 dB, which is much higher than that of the homogeneous composites prepared by the direct blending. Fan et al. [107] prepared the silver nanosheets (AgNS)/epoxy composites with porous structures by the direct blending and supercritical CO_2_ foaming technique, using AgNS as the conductive filler and epoxy resin as the matrix (Fig. 8b). When the AgNS content is 20 wt%, the EMI SE of the porous AgNS/epoxy composites reaches 42 dB, which is much higher than that of the composites with the homogeneous structure prepared by direct blending. The above researches show that the foaming method for preparing the EMI shielding composites are suitable for a variety of polymer matrices, and the conductive fillers can be dispersed again during the foaming process. However, the improvement of EMI SE for polymer matrix composites by a single foaming technique is limited, which is far from that of metal materials. In practical applications, it is also necessary to combine other methods to further improve the EMI shielding performance of the composites.

#### Sol–gel Method

**Fig. 9:**
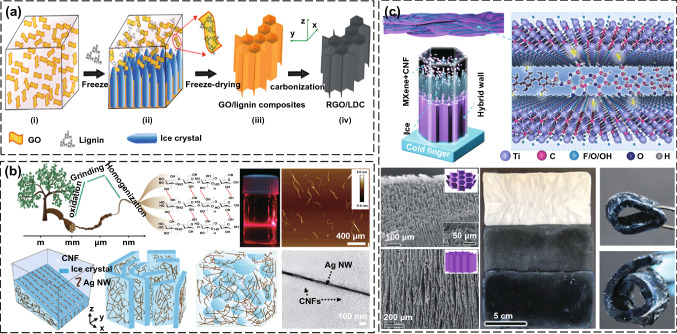
Schematic illustrating the fabrication, SEM images and digital photos of **a** RGO/LDC composites, **b** AgNW/CNF aerogels, and **c** MXene/CNF aerogels [110–112]. Copyright © 2018 American Chemical Society. Copyright © 2020 Wiley–VCH. Copyright © 2020 American Chemical Society

The sol–gel method used to prepare porous polymer matrix EMI shielding composites is very common in laboratory research. Usually, the highly active conductive fillers and polymer monomers are uniformly mixed in the liquid phase to form a stable sol system. The sol forms a gel with three-dimensional network structure in solution by hydrolysis reaction, condensation reaction and hydrogen bonding. The gel is freeze-dried to obtain porous polymer matrix EMI shielding composites [108]. Zeng et al. [109] uniformly mixed the MWCNTs and waterborne polyurethane (WPU) in an aqueous solution to form a stable sol system, which was directly freeze-dried to obtain the MWCNTs/WPU aerogels with an anisotropic porous structure. The results show that the EMI SE of the MWCNTs/WPU aerogels reaches 50 dB at a density of 126 mg/cm^3^. The excellent EMI shielding performance is attributed to the highly conductive network of MWCNTs, the anisotropic porous structure and the polarization effect between MWCNTs and WPU matrix. Lu et al. [110] first mixed the GO and lignin homogeneously in an aqueous solution to form a stable sol–gel system. Then the system was unidirectionally freeze-dried to prepare the ultralight GO/lignin aerogels with micro-channel. Finally, the aerogels were thermally reduced to obtain the reduced GO/lignin-derived carbon (RGO/LDC) composites (Fig. 9a). The introduction of lignin enhances the interfacial polarization effect and the absorption of electromagnetic waves in the RGO/LDC composites. The obtained composites achieve an EMI SE of 49 dB at an ultra-low density of 8.0 mg cm^−3^, which is higher than that of a single-phase RGO foam. Nyström et al. [111, 112] dispersed the AgNW and MXene in the CNF sol system, and then directly freeze-dried to prepare the AgNW/CNF and MXene/CNF aerogels, respectively (Fig. 9b, c). The EMI SE of the AgNW/CNF aerogel reaches 40 dB at a density of 1.7 mg cm^−3^, while that of the MXene/CNF aerogel reaches 35 dB at a density of 1.5 mg cm^−3^. The above researches show that the polymer matrix composites prepared by the sol–gel method achieve a rapid increase in EMI SE per unit density due to their internal porous structure. However, the polymer matrix networks prepared by this method have weak connection strength, resulting in poor mechanical properties of the composites [113, 114].

#### Template Method

**Fig. 10:**
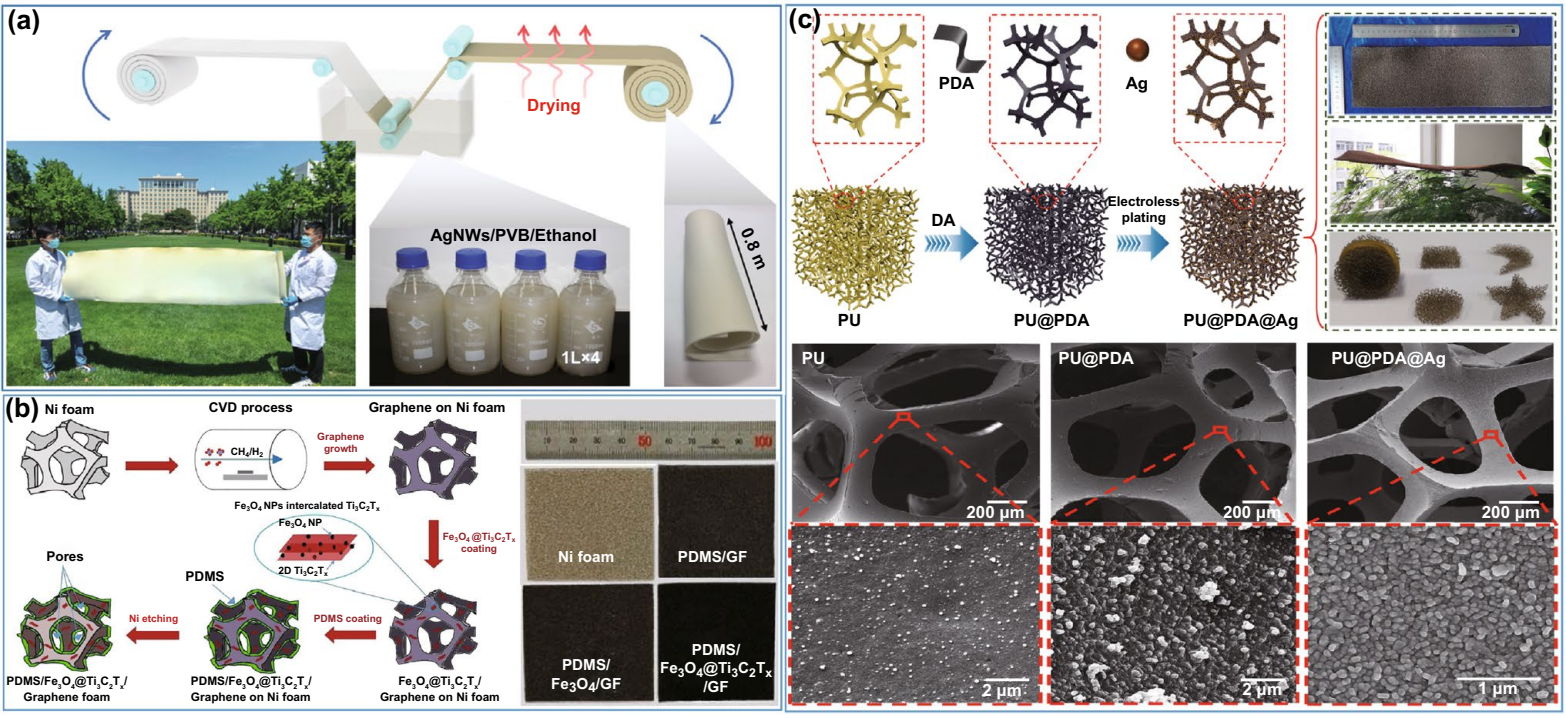
Schematic illustrating the fabrication and SEM images of **a** AgNW/PVB/MS sponges, **b** Fe_3_O_4_@MXene/GF/PDMS foams, and **c** PU@PDA@Ag sponges [116–118]. Copyright © 2019 Wiley–VCH. Copyright © 2020 Elsevier

The template method refers to the preparation of porous polymer matrix EMI shielding composites by uniformly attaching conductive fillers to the surface of porous polymer matrix skeletons through chemical plating, chemical vapor deposition (CVD), electrostatic adsorption, etc. The conductive fillers are oriented and continuously distributed along the polymer matrix porous framework, which helps to form a conductive network efficiently, thereby improving the EMI shielding performance of the composites [115]. Lin et al. [116] immersed the melamine sponge (MS) in the AgNW/polyvinyl butyral (PVB) solution so that the AgNW/PVB was evenly coated on the surface of the MS framework, which was dried to obtain the AgNW/PVB/MS sponge (Fig. 10a). The EMI SE of the AgNW/PVB/MS sponge with a thickness of 5 mm reaches 60 dB, which is higher than that of a commercial EMI shielding sponge with the same thickness. Nguyen et al. [117] successively deposited the graphene (GF), Fe_3_O_4_@MXene and PDMS on the surface of the nickel foam template through a CVD process. The Fe_3_O_4_@MXene/GF/PDMS foam was obtained after the nickel foam template was etched by a ferric chloride solution (Fig. 10b). The EMI SE of Fe_3_O_4_@MXene/GF/PDMS foam is 80 and 77 dB in X-band and Ku-band, respectively. Gu et al. [118] reported the PU@PDA@Ag sponge by a bio-response template method, which could be obtained by two steps: (i) PDA was decorated on the surface of PU sponge by dopamine self-polymerization. (ii) Ag nanoparticles were in-situ grown on the surface of PU sponge by electroless plating. The EMI SE of the obtained PU@PDA@Ag sponge is as high as 84 dB at a thickness of 5 mm (Fig. 10c). The above researches show that the advantages of template method include the low density, the simplicity of preparation process, and the possibility of large-scale preparation. However, if the porous composites prepared by template method are deformed during applications, the conductive fillers are easily detached from the polymer matrix skeleton, which will affect the EMI shielding lifetime of the composites [119, 120].

### Layered Polymer Matrix EMI Shielding Composites

The layered polymer matrix EMI shielding composites are a class of materials with a layered structure. The electromagnetic waves can be reflected for multiple times inside the composites due to the layered structure, thereby improving their EMI shielding performance greatly. The lightweight and flexible polymer matrix EMI shielding films are the most representative type of material with a layered structure, and has attracted wide attention from researchers. At present, the preparation of polymer matrix EMI shielding films is mainly through vacuum filtration method, coating method and polymer-assisted method [121–123].

#### Vacuum Filtration Method

**Fig. 11:**
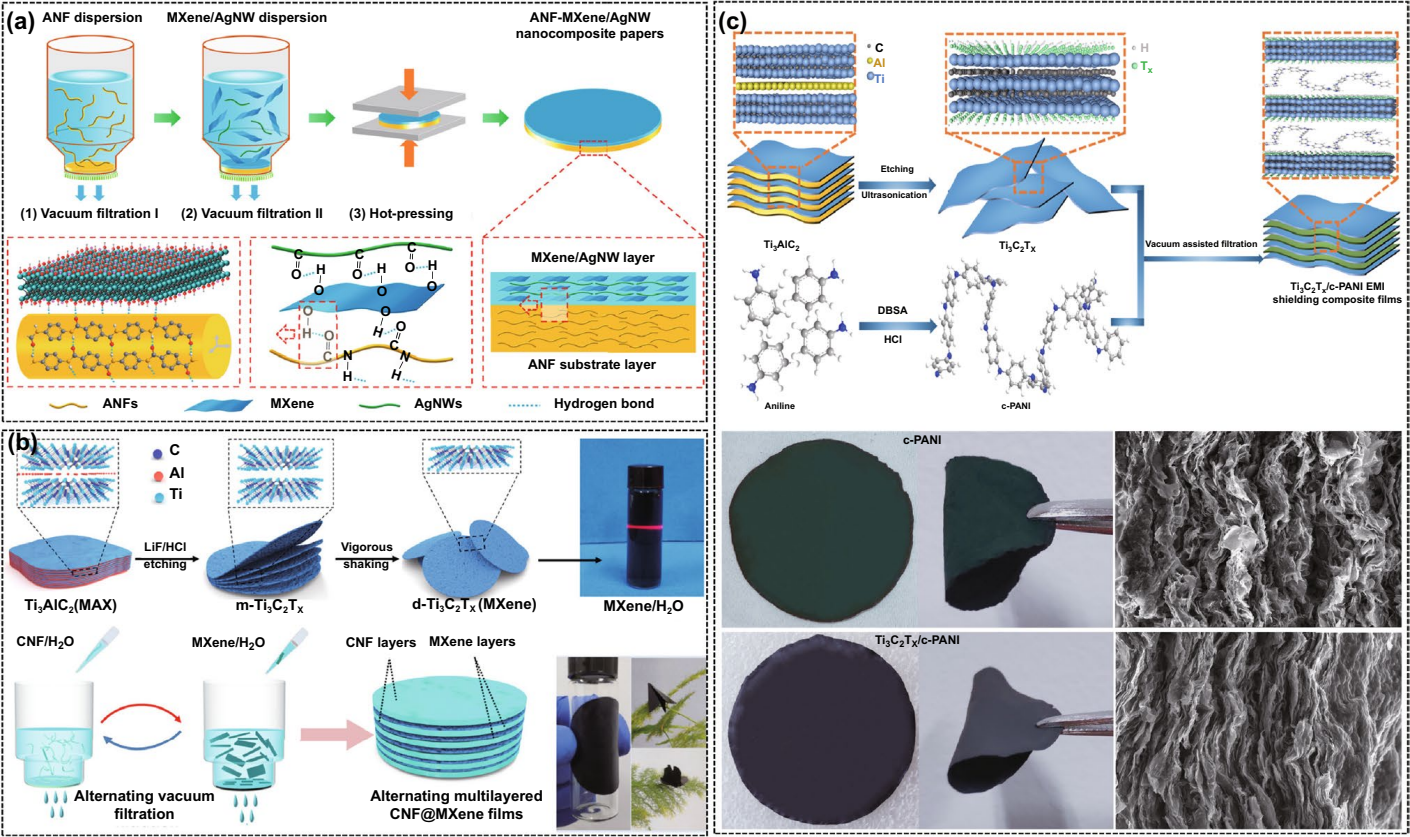
Schematic illustrating the fabrication and SEM images of **a** ANF-MXene/AgNW, **b** CNF@MXene, and **c** MXene/c-PANI composite films [124–126]. Copyright © 2020 American Chemical Society. Copyright © 2019 Elsevier

The vacuum filtration method refers to prepare the polymer matrix EMI shielding films by removing the solvent in the mixed solution of conductive fillers and polymer matrix under pressure difference. The films prepared by the vacuum filtration method have an obvious layered structure and can efficiently reflect the electromagnetic waves for multiple times. Ma et al. [124] used the two-dimensional MXene and one-dimensional AgNWs as the conductive functional layer and aramid nanofibers (ANFs) as the high-performance enhancement layer to fabricate the flexible and high-strength ANF-MXene/AgNW composite films with a bilayer structure by the vacuum-assisted filtration and hot-press molding techniques (Fig. 11a). This work provides theoretical and technical guidance for the design and fabrication of flexible and high-strength layered polymer EMI shielding composites. The EMI SE of the ANF-MXene/AgNW films with a thickness of 0.091 mm is about 80 dB in the X-band, which is higher than that of the films with a homogeneous structure with the same MXene/AgNW content. Zhou et al. [125] prepared the CNF@MXene multilayer films with an alternating structure by an alternating vacuum filtration method using CNF and MXene as raw materials (Fig. 11b). The EMI SE of the CNF@MXene multilayer films with a thickness of 0.035 mm in the X-band is about 40 dB, which is higher than that of the films with a homogeneous structure. The main reason is that the alternating multilayer structure of CNF@MXene films increases the "zigzag" reflection mechanism of electromagnetic waves. Gu et al. [126] first co-doped the modified polyaniline (c-PANI) with dodecyl benzene sulfonic acid and hydrochloric acid, then prepared less layers of MXene using the ionic intercalation and ultrasound-assisted techniques, and finally prepared the MXene/c-PANI EMI shielding films by a vacuum filtration method (Fig. 11c). The *σ* and EMI SE of the MXene/c-PANI EMI shielding films with a thickness of 0.040 mm are 24.4 S cm^−1^ and 36 dB, respectively, which are 81 times and 2.3 times higher than those of the pure c-PANI films. It can be seen that the thickness of the EMI shielding films prepared by vacuum filtration method is easy to control and the utilization rate of raw materials is relatively high. However, the area of the EMI shielding films is restricted by the size of the equipment, and the system containing two-dimensional sheet materials takes a long time to form films by vacuum filtration [127, 128].

#### Coating Method

**Fig. 12:**
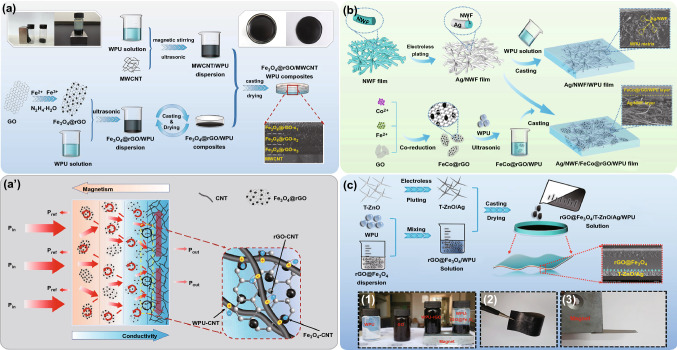
Schematic illustrating the fabrication of **a** Fe_3_O_4_@rGO/MWCNTs/WPU, **b** CNF@MXene, and **c** Ti_3_C_2_T_x_/c-PANI composite films [40, 130, 131]. Copyright © 2019 Elsevier. Copyright © 2020 Elsevier. Copyright © 2018 American Chemical Society

The coating method refers to the preparation of polymer matrix EMI shielding films by curing a mixed system containing conductive fillers and resin matrix in an appropriate mold. Obviously, the coating method is relatively simple and widely used in actual production. In order to build a more complete conductive network inside the EMI shielding films, the viscosity and curing time of the resin matrix are the keys to the process. Sheng et al. [40] constructed the ordered multilayer Fe_3_O_4_@rGO/MWCNTs/WPU films by the layer-by-layer coating method. The Fe_3_O_4_@rGO and MWCNTs provide negative magnetic gradient and positive electrical conductivity gradient, respectively (Fig. 12a). The EMI SE of Fe_3_O_4_@rGO/MWCNTs/WPU films reaches 35.9 dB when the MWCNT content is 60%. Jin et al. [129] prepared the multilayer PVA/MXene films by alternate coating using PVA and MXene as raw materials. The continuous MXene layers provide the compact networks for conducting electrons, allowing the multilayer PVA/MXene films to exhibit excellent EMI shielding performance. The PVA/Mxene films with 27 μm thickness exhibits the *σ* of 716 S m^−1^ and the maximum EMI SE of 44.4 dB when the MXene content is 19.5 wt%. Ren et al. [130] first prepared the FeCo@rGO/WPU hybrid system using the reduced graphene oxide grafted with FeCo alloy (FeCo@rGO) as filler and WPU as matrix, and then applied the hybrid system to the encapsulation of silver-plated nonwoven fabric (Ag/NWF) by coating method to obtain the Ag/NWF/FeCo@rGO/WPU films (Fig. 12b). The EMI SE of the Ag/NWF/FeCo@rGO/WPU films reaches 77 dB when the Ag and FeCo@rGO contents are 10.5 wt% and 10 wt%, respectively. Xu et al. [131] prepared the Fe_3_O_4_@rGO/T-ZnO/Ag/WPU films by the coating method using Fe_3_O_4_@rGO and silver-plated tetra-needle ZnO whiskers (T-ZnO/Ag) as fillers and WPU as matrix (Fig. 12c). Because of the differences of Fe_3_O_4_@rGO and T-ZnO/Ag in density, a gradient structure is automatically formed during the film formation process. The Fe_3_O_4_@rGO is uniformly distributed throughout the thickness range of the film, forming the effective three-dimensional electromagnetic wave absorption network. The T-ZnO/Ag is uniformly deposited at the bottom of the film, forming the effective two-dimensional electromagnetic wave reflection network. When electromagnetic waves penetrate the Fe_3_O_4_@rGO/T-ZnO/Ag/WPU films, its specific structure can trigger the "absorption-reflection-reabsorption" mechanism. The EMI SE of the Fe_3_O_4_@rGO/T-ZnO/Ag/WPU films with a thickness of 0.5 mm reaches 87 dB under the contents of 0.8 vol% Fe_3_O_4_@rGO and 5.7 vol% T-ZnO/Ag. The above researches show that the EMI shielding films prepared by the coating method are not restricted by the equipment and can be produced on a large scale. The EMI shielding films with a gradient structure can be prepared by using the density difference of the conductive fillers, thereby making the films have excellent electromagnetic wave absorption performance. However, the coating method also faces some problems, such as the time-consuming curing process of the resin matrix, and the interlayer of the films are not dense [132–134].

#### Polymer-assisted Method

**Fig. 13:**
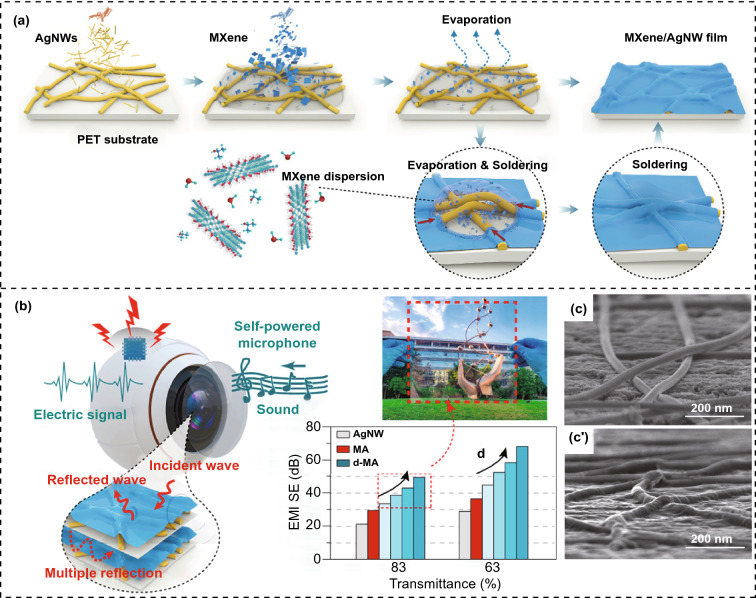
**a** Schematic illustrating the fabrication, **b** EMI SE, and **c** SEM images of MXene/AgNW/PET composite films [140]. Copyright © 2020 American Chemical Society

The polymer-assisted method refers to the direct deposition of conductive fillers on the surface of the polymer matrix films through laser printing, high-pressure spraying, sputtering deposition, etc., to prepare the polymer matrix EMI shielding films. The method is the main technical tool for the preparation of transparent EMI shielding films [30, 135, 136]. Wang et al. [137] used the sputtering deposition technique to deposit copper-doped silver and indium tin oxide (ITO) films sequentially on a polyethylene terephthalate (PET) film substrate. The obtained Cu-Ag/ITO/PET films transmit 96.5% of visible light and show an EMI SE of 26 dB over the wide frequency range of 32 GHz. Gu et al. [138] first constructed a regular AgNW grid on the glass surface by a laser printing technique, and then transferred the AgNW grid to the PVA/PET substrate by a printing technique, and finally prepared the AgNW/PVA/PET films by a hot pressing. The resultant films obtain an EMI SE of 44 dB and a visible light transmittance of 67.8%. Zhu et al. [139] first coated the AgNW network on the glass substrate by the Meyer rod coating method, and then encapsulated the AgNW network through poly dimethyl diallyl ammonium chloride (PDDA) to obtain the AgNW/PDDA films. The EMI SE and visible light transmittance of the resultant films are 31 dB and 91.3%, respectively. Chen et al. [140] successively constructed the dense AgNW and MXene conductive grids on PET substrates using the high-pressure air-assisted spraying technology (Fig. 13). The EMI SE and visible light transmittance of the obtained films can reach 49 dB and 83%, respectively. Although the polymer-assisted method can quickly and continuously prepare composite films, the connection strength between the conductive filler network and the polymer matrix is not high, which easily leads to interface separation [141].

### Preformed Polymer Matrix EMI Shielding Composites

**Fig. 14:**
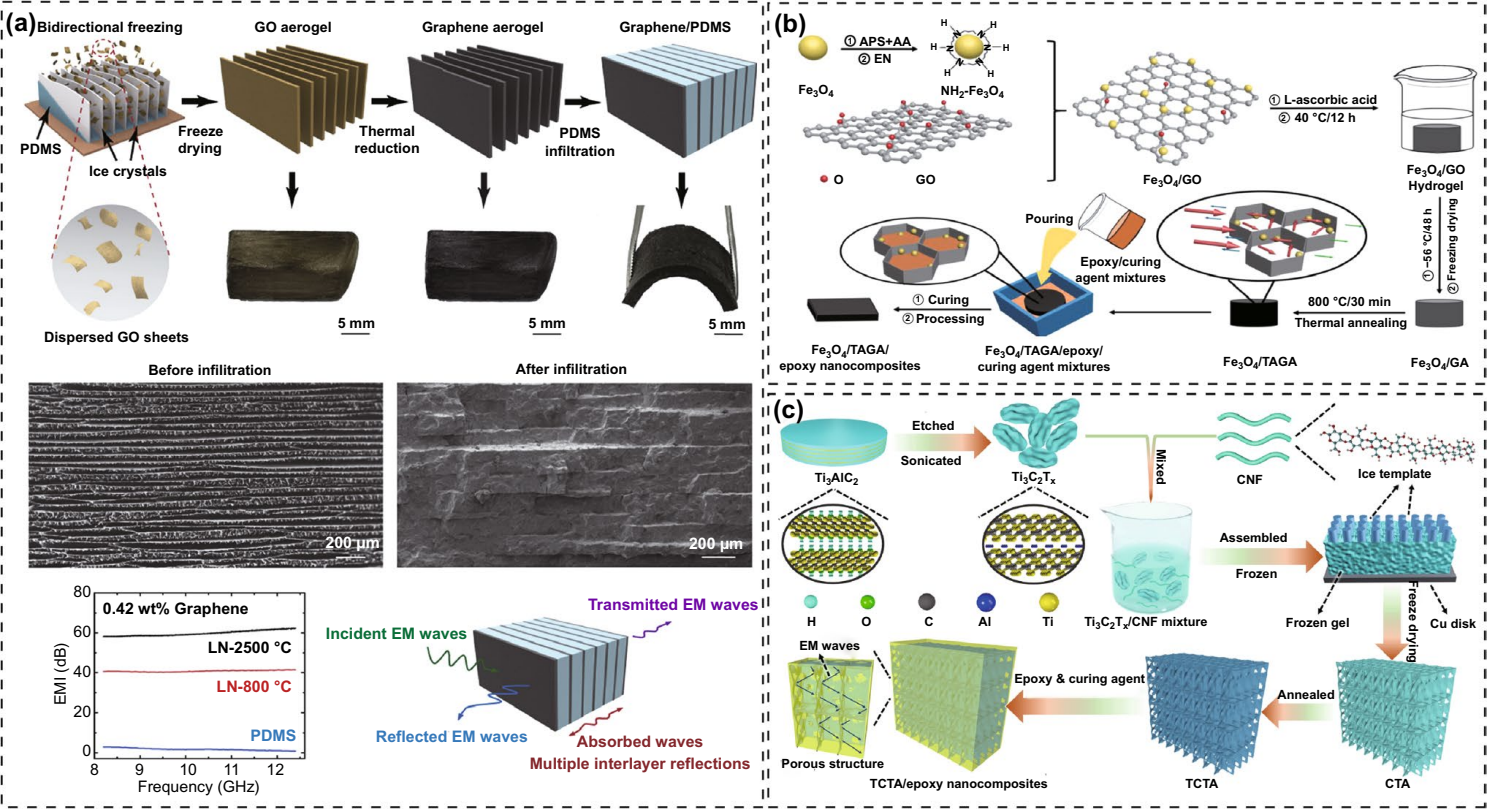
Schematic illustrating the fabrication, SEM images and EMI SE of **a** graphene/PDMS, **b** Fe_3_O_4_/TAGA/epoxy, and **c** TCTA/epoxy composites [146, 149, 150]. Copyright © 2019 Elsevier. Copyright © 2018 Elsevier

The preformed polymer matrix EMI shielding composites are a class of materials in which the conductive fillers are pre-constructed in the resin matrix with a specific three-dimensional structure by freeze-drying, hydrothermal, or CVD methods [142, 143]. The conductive fillers in the preformed composites have formed a stable three-dimensional conductive framework, and the polymer matrix with very low viscosity does not destroy the original conductive network during the backfilling process. Therefore, the composites can achieve rapid improvement of EMI SE with low contents of conductive fillers [144, 145]. Gao et al. [146] prepared a biaxially arranged graphene network with a laminar structure by bi-directional freezing technique, which was thermally reduced at 2500 °C and impregnated with PDMS to obtain the graphene/PDMS composites with a mother-of-pearl-like structure (Fig. 14a). When the graphene content is 0.42 wt%, the EMI SE values of the graphene/PDMS composites in the parallel and perpendicular directions to the graphene plane reach 16 and 42 dB, respectively. Fang et al. [147] first prepared the graphene foam (GF) by a CVD method using nickel foam as a template, then grew Fe_3_O_4_ in situ on the surface of GF to obtain GF/h-Fe_3_O_4_, and finally obtained the GF/h-Fe_3_O_4_/PDMS composites by impregnating the GF/h-Fe_3_O_4_ with PDMS. The composites exhibit an EMI SE of 70 dB when the GF/h-Fe_3_O_4_ content is 12 wt%. Shen et al. [148] prepared the carbon skeleton with a continuous structure by carbonizing the natural wood, which was backfilled with epoxy resin to prepare the epoxy/wood-derived carbon composites. The *σ* and EMI SE of the obtained composites are 12.5 S m^−1^ and 27.8 dB, respectively, when the carbon content is 7.0 vol%. In our previous works, a series of preformed polymer matrix EMI shielding composites with the controllable structure were prepared. Gu et al. [149] first prepared the homogeneous hybrid system of amino-functionalized Fe_3_O_4_ (NH_2_-Fe_3_O_4_) nanoparticles, GO and L-ascorbic acid, then the hybrid system was hydrothermally reacted to obtain the Fe_3_O_4_/graphene aerogel (Fe_3_O_4_/GA), which was thermally annealed and backfilled with epoxy resin to obtain the Fe_3_O_4_/thermally annealed GA (Fe_3_O_4_/TAGA)/epoxy composites (Fig. 14b). When the mass ratio of GO to NH_2_-Fe_3_O_4_ is 2:1 and the total Fe_3_O_4_/TAGA content is 2.7 wt%, the EMI SE of the resultant composites reaches 35 dB, which is much higher than that of the epoxy composites prepared by the direct blending method with the same fillers content. Furthermore, Gu et al. [150] prepared the anisotropic and highly conductive CNF/MXene aerogel (CTA) based on the unidirectional freeze-drying technique, and obtained the thermally reduced CTA (TCTA)/epoxy composites after the thermal reduction-vacuum-assisted impregnation (Fig. 14c). The results show that the obtained composites have the complete and efficient three-dimensional conductive network and the low percolation threshold. When TCTA fraction is 1.38 vol%, the radial *σ* and axial EMI SE of composites are 1672 S m^−1^ and 74 dB, respectively, which are much higher than those of epoxy composites prepared by the direct blending method with the same TCTA content. The above researches show that the multiple reflection ability of the composites for electromagnetic waves can be improved by pre-constructing a stable three-dimensional conductive network skeleton in the resin matrix, which in turn enhances the EMI shielding performance of the composites. However, the preconstruction of a three-dimensional conductive network skeleton in the resin matrix also faces some problems, such as poor interfacial compatibility between the three-dimensional skeleton structure and the resin matrix, and the continuous phase of the fillers hinders the continuous phase structure of the resin matrix. These problems eventually lead to the weak mechanical strength of the composites [108, 151, 152].

## Conclusions and Outlook

This review first discusses the key concepts, loss mechanisms and test methods of EMI shielding. Then, the current development status of EMI shielding materials is totally summarized, and the research progress of polymer matrix EMI shielding composites with different structures is detailedly illustrated, in which their preparation methods and corresponding evaluations are highlighted. It is worth noting that the purpose of the preparation methods for all polymer matrix EMI shielding composites with different structures is to build more complete conductive networks and more conductive-insulating interfaces, thereby achieving stronger polarization loss and conduction loss. Although a series of favorable advances have been made in the preparation of polymer matrix EMI shielding composites, there are still many problems. The details are summarized as follows:

1. Although the homogeneous polymer matrix EMI shielding composites are simple to process, they require a high content of conductive fillers to build an effective conductive network in the polymer matrix, and to ensure that their EMI SE is higher than the industrial application standard of 20 dB. However, the high content of conductive fillers will cause the decrease of mechanical properties and processing difficulties. The homogeneous polymer matrix EMI shielding composites are suitable for applications where the EMI shielding performance requirement is not high, while the mass-production and application are needed.
2. The conductive fillers in the segregated polymer matrix EMI shielding composites are confined in the interfaces of the polymer micro-zones. The probability of interlap between the conductive fillers is significantly increased, thereby enhancing the EMI shielding performance of the composites. However, the conductive fillers form a single continuous network structure inside the composites, and the polymer particles tend to exist in isolation, which will result in relatively poor mechanical properties. This type of EMI shielding composites can be applied to the non-load-bearing parts that require high EMI shielding performances.
3. The porous polymer matrix EMI shielding composites have the advantages of low density and good toughness. However, the polymer matrix used for the preparation of porous composites is mostly concentrated in PU, MS, PDMS, WPU, etc., which have poor heat resistance and mechanical strength. These problems limit the applications of porous composites in high-density and high-integration electronic components. As a representative of lightweight EMI shielding materials, they have great potentials for sandwich parts in areas of aircraft, military engineering and automobile industry.
4. The layered polymer matrix EMI shielding composites can induce multiple reflections of electromagnetic waves between the internal layers due to unique stratified structure. However, the layered polymer matrix EMI shielding composites prepared based on vacuum filtration, layered coating, and polymer-assisted methods have weak interface connection strength and are time-consuming. The ultra-thin structure makes the layered polymer matrix EMI shielding composites gain widespread attention in the fields of visual shielding windows, microelectronic devices and electronic communication equipment.
5. The preformed polymer matrix EMI shielding composites have the advantages such as low content of conductive fillers, high overlap efficiency of the conductive network and excellent EMI shielding performance. However, a series of preformed polymer matrix EMI shielding composites developed based on freeze-drying, hydrothermal, and CVD methods are facing problems such as scarcity of candidate conductive fillers, harsh preparation conditions and long reaction cycles. This type of EMI shielding composites is expected to be used in small-scale equipment components that require superior EMI shielding performance.

Therefore, the development of next-generation high-performance polymer matrix EMI shielding composites based on the reasonable structural design and new preparation methods has become a key research direction, which is necessary to promote the full replacement of metallic EMI shielding materials. It is believed that in the near future, polymer matrix EMI shielding composites will be more widely and comprehensively applied in various fields including aerospace, automobile manufacturing, artificial intelligence and precision instruments.

## References

[1] Thomassin JM, Jerome C, Pardoen T, Bailly C, Huynen I (2013). Polymer/carbon based composites as electromagnetic interference (EMI) shielding materials. Mater. Sci. Eng. R-Rep..

[2] Shahzad F, Alhabeb M, Hatter CB, Anasori B, Hong SM (2016). Electromagnetic interference shielding with 2D transition metal carbides (MXenes). Science.

[3] Abbasi H, Antunes M, Velasco JI (2019). Recent advances in carbon-based polymer nanocomposites for electromagnetic interference shielding. Prog. Mater. Sci..

[4] Song P, Liu B, Liang C, Ruan K, Qiu H (2021). Lightweight, flexible cellulose-derived carbon aerogel@reduced graphene oxide/PDMS composites with outstanding EMI shielding performances and excellent thermal conductivities. Nano-Micro Lett..

[5] Liu S, Liu J, Dong X, Duan Y (2013). Electromagnetic Wave Shielding and Absorbing Materials.

[6] Yang R, Gui X, Yao L, Hu Q, Yang L (2021). Ultrathin, lightweight, and flexible CNT buckypaper enhanced using MXenes for electromagnetic interference shielding. Nano-Micro Lett..

[7] Kumar P, Maiti UN, Sikdar A, Das TK, Kumar A (2019). Recent advances in polymer and polymer composites for electromagnetic interference shielding: review and future prospects. Polym. Rev..

[8] Wu L, Wu F, Sun Q, Shi J, Xie A (2021). A TTF–TCNQ complex: an organic charge-transfer system with extraordinary electromagnetic response behavior. J. Mater. Chem. C.

[9] Jiang DW, Murugadoss V, Wang Y, Lin J, Ding T (2019). Electromagnetic interference shielding polymers and nanocomposites: a review. Polym. Rev..

[10] Guo M, Huang J, Deng Y, Shen H, Ma Y (2015). pH-Responsive cyanine-grafted graphene oxide for fluorescence resonance energy transfer-enhanced photothermal therapy. Adv. Funct. Mater..

[11] L. Wang, Z. Ma, L. Chen, Y. Zhang, D. Cao, et al., Polymer-based EMI shielding composites with 3D conductive networks: a mini-review. SusMat (2021) doi:10.1002/sus2.21

[12] Iqbal A, Sambyal P, Koo CM (2020). 2D MXenes for electromagnetic shielding: a review. Adv. Funct. Mater..

[13] Yang X, Fan S, Li Y, Guo Y, Li Y (2020). Synchronously improved electromagnetic interference shielding and thermal conductivity for epoxy nanocomposites by constructing 3D copper nanowires/thermally annealed graphene aerogel framework. Compos. A-Appl. S..

[14] Singh AK, Shishkin A, Koppel T, Gupta N (2018). A review of porous lightweight composite materials for electromagnetic interference shielding. Compos. B-Eng..

[15] Guo Y, Pan L, Yang X, Ruan K, Han Y (2019). Simultaneous improvement of thermal conductivities and electromagnetic interference shielding performances in polystyrene composites via constructing interconnection oriented networks based on electrospinning technology. Compos. A Appl. S..

[16] Pan F, Liu Z, Deng B, Dong Y, Zhu X (2021). Lotus leaf-derived gradient hierarchical porous C/MoS_2_ morphology genetic composites with wideband and tunable electromagnetic absorption performance. Nano-Micro Lett..

[17] Song P, Liu B, Qiu H, Shi X, Cao D (2021). MXenes for polymer matrix electromagnetic interference shielding composites: a review. Compos. Commun..

[18] Zhu Y, Liu J, Guo T, Wang JJ, Tang X (2021). Multifunctional Ti_3_C_2_T_x_ MXene composite hydrogels with strain sensitivity toward absorption-dominated electromagnetic-interference shielding. ACS Nano.

[19] Zhang Y, Ruan K, Shi X, Qiu H, Pan Y (2021). Ti_3_C_2_T_x_/rGO porous composite films with superior electromagnetic interference shielding performances. Carbon.

[20] Sankaran S, Deshmukh K, Ahamed MB, Pasha SKK (2018). Recent advances in electromagnetic interference shielding properties of metal and carbon filler reinforced flexible polymer composites: a review. Compos. A Appl. S..

[21] Pakdel E, Wang J, Kashi S, Sun L, Wang X (2020). Advances in photocatalytic self-cleaning, superhydrophobic and electromagnetic interference shielding textile treatments. Adv. Colloid Interface Sci..

[22] Nazir A, Yu HJ, Wang L, Haroon M, Ullah RS (2018). Recent progress in the modification of carbon materials and their application in composites for electromagnetic interference shielding. J. Mater. Sci..

[23] Jiao Y, Cheng S, Wu F, Pan X, Xie A (2021). MOF-Guest complex derived Cu/C nanocomposites with multiple heterogeneous interfaces for excellent electromagnetic waves absorption. Compos. B Eng..

[24] Geetha S, Kumar KKS, Rao CRK, Vijayan M, Trivedi DC (2009). EMI shielding: methods and materials: a review. J. Appl. Polym. Sci..

[25] Cheng S, Xie A, Pan X, Zhang K, Zhang C (2021). Modulating surficial oxygen vacancy of the VO_2_ nanostructure to boost its electromagnetic absorption performance. J. Mater. Chem. C.

[26] Huang W, Zhang X, Zhao Y, Zhang J, Liu P (2020). Hollow N-doped carbon polyhedra embedded Co and Mo_2_C nanoparticles for high-efficiency and wideband microwave absorption. Carbon.

[27] Song P, Qiu H, Wang L, Liu X, Zhang Y (2020). Honeycomb structural rGO-MXene/epoxy nanocomposites for superior electromagnetic interference shielding performance. Sustain. Mater. Technol..

[28] Zhao J, Lu Y, Ye W, Wang L, Liu B (2019). Enhanced wave-absorbing performances of silicone rubber composites by incorporating C-SnO_2_-MWCNT absorbent with ternary heterostructure. Ceram. Int..

[29] Bagotia N, Choudhary V, Sharma DK (2018). A review on the mechanical, electrical and EMI shielding properties of carbon nanotubes and graphene reinforced polycarbonate nanocomposites. Polym. Adv. Technol..

[30] Zeng Z, Jiang F, Yue Y, Han D, Lin L (2020). Flexible and ultrathin waterproof cellular membranes based on high-conjunction metal-wrapped polymer nanofibers for electromagnetic interference shielding. Adv. Mater..

[31] Song Q, Chen B, Zhou Z, Lu C (2021). Flexible, stretchable and magnetic Fe_3_O_4_@Ti_3_C_2_T_x_/elastomer with supramolecular interfacial crosslinking for enhancing mechanical and electromagnetic interference shielding performance. Sci. China Mater..

[32] Choi HK, Lee A, Park M, Lee DS, Bae S (2021). Hierarchical porous film with layer-by-layer assembly of 2D copper nanosheets for ultimate electromagnetic interference shielding. ACS Nano.

[33] Park J, Lee JW, Choi HJ, Jang WG, Kim TS (2019). Electromagnetic interference shielding effectiveness of sputtered NiFe/Cu multi-layer thin film at high frequencies. Thin Solid Films.

[34] Sambyal P, Noh SJ, Hong JP, Kim WN, Iqbal A (2019). FeSiAl/metal core shell hybrid composite with high-performance electromagnetic interference shielding. Compos. Sci. Technol..

[35] Song K, Pan FS, Chen XH, Zhang ZH, Tang AT (2015). Effect of texture on the electromagnetic shielding property of magnesium alloy. Mater. Lett..

[36] Jia Z, Zhang M, Liu B, Wang F, Wei G (2020). Graphene foams for electromagnetic interference shielding: a review. ACS Appl. Nano Mater..

[37] Pradhan SS, Unnikrishnan L, Mohanty S, Nayak SK (2020). Thermally conducting polymer composites with EMI shielding: a review. J. Electron. Mater..

[38] Zhao J, Zhang J, Wang L, Lyu S, Ye W (2020). Fabrication and investigation on ternary heterogeneous MWCNT@TiO_2_-C fillers and their silicone rubber wave-absorbing composites. Compos. A Appl. S..

[39] Massa A, Rocca P, Oliveri G (2015). Compressive sensing in electromagnetics: a review. IEEE Antenn. Propag. M..

[40] Sheng A, Ren W, Yang Y, Yan D-X, Duan H (2020). Multilayer WPU conductive composites with controllable electro-magnetic gradient for absorption-dominated electromagnetic interference shielding. Compos. A Appl. S..

[41] Park J-B, Rho H, Cha A-N, Bae H, Lee SH (2020). Transparent carbon nanotube web structures with Ni-Pd nanoparticles for electromagnetic interference (EMI) shielding of advanced display devices. Appl. Surf. Sci..

[42] Iqbal A, Shahzad F, Hantanasirisakul K, Kim M-K, Kwon J (2020). Anomalous absorption of electromagnetic waves by 2D transition metal carbonitride Ti_3_CNT_x_ (MXene). Science.

[43] Fei Y, Liang M, Yan L, Chen Y, Zou H (2020). Co/C@cellulose nanofiber aerogel derived from metal-organic frameworks for highly efficient electromagnetic interference shielding. Chem. Eng. J..

[44] Ren F, Song DP, Li Z, Jia LC, Zhao YC (2018). Synergistic effect of graphene nanosheets and carbonyl iron-nickel alloy hybrid filler on electromagnetic interference shielding and thermal conductivity of cyanate ester composites. J. Mater. Chem. C.

[45] Luo J, Huo L, Wang L, Huang X, Li J (2020). Superhydrophobic and multi-responsive fabric composite with excellent electro-photo-thermal effect and electromagnetic interference shielding performance. Chem. Eng. J..

[46] Liu QH, Cao Q, Bi H, Liang CY, Yuan KP (2016). CoNi@SiO_2_@TiO_2_ and CoNi@Air@TiO_2_ Microspheres with strong wideband microwave absorption. Adv. Mater..

[47] Liang XH, Man ZM, Quan B, Zheng J, Gu WH (2020). Environment-stable Co_x_Ni_y_ encapsulation in stacked porous carbon nanosheets for enhanced microwave absorption. Nano-Micro Lett..

[48] Zhang DQ, Xiong YF, Cheng JY, Chai JX, Liu TT (2020). Synergetic dielectric loss and magnetic loss towards superior microwave absorption through hybridization of few-layer WS_2_ nanosheets with NiO nanoparticles. Sci. Bull..

[49] Shi H, Liu C, Jiang Q, Xu J (2015). Effective approaches to improve the electrical conductivity of PEDOT:PSS: a review. Adv. Electron. Mater..

[50] Zhang X, Zhao N, He C (2020). The superior mechanical and physical properties of nanocarbon reinforced bulk composites achieved by architecture design: a review. Prog. Mater. Sci..

[51] Zhao J, Zhang J, Wang L, Li J, Feng T (2020). Superior wave-absorbing performances of silicone rubber composites via introducing covalently bonded SnO_2_@MWCNT absorbent with encapsulation structure. Compos. Commun..

[52] Liu H, Li Q, Zhang S, Yin R, Liu X (2018). Electrically conductive polymer composites for smart flexible strain sensors: a critical review. J. Mater. Chem. C.

[53] Liang C, Qiu H, Song P, Shi X, Kong J (2020). Ultra-light MXene aerogel/wood-derived porous carbon composites with wall-like “mortar/brick” structures for electromagnetic interference shielding. Sci. Bull..

[54] Wang L, Shi X, Zhang J, Zhang Y, Gu J (2020). Lightweight and robust rGO/sugarcane derived hybrid carbon foams with outstanding EMI shielding performance. J. Mater. Sci. Technol..

[55] Wang X, Yang C, Jin J, Li X, Cheng Q (2018). High-performance stretchable supercapacitors based on intrinsically stretchable acrylate rubber/MWCNTs@conductive polymer composite electrodes. J. Mater. Chem. A.

[56] Qiu M, Zhang Y, Wen B (2018). Facile synthesis of polyaniline nanostructures with effective electromagnetic interference shielding performance. J. Mater. Sci. Mater. El..

[57] Mueller D, Rambo CR, Recouvreux DOS, Porto LM, Barra GMO (2011). Chemical in situ polymerization of polypyrrole on bacterial cellulose nanofibers. Synth. Met..

[58] Wang Y, Zhu C, Pfattner R, Yan H, Jin L (2017). A highly stretchable, transparent, and conductive polymer. Sci. Adv..

[59] Han P, Zhang X, Qiao J (2016). Intrinsically conductive polymer fibers from thermoplastic trans-1,4-polyisoprene. Langmuir.

[60] Li J, Wang A, Qin J, Zhang H, Ma Z (2021). Lightweight polymethacrylimide@copper/nickel composite foams for electromagnetic shielding and monopole antennas. Compos. A Appl. S..

[61] Ma C, Cao WT, Zhang W, Ma MG, Sun WM (2021). Wearable, ultrathin and transparent bacterial celluloses/MXene film with Janus structure and excellent mechanical property for electromagnetic interference shielding. Chem. Eng. J..

[62] Li L, Zhao S, Luo X-J, Zhang H-B, Yu Z-Z (2021). Smart MXene-based Janus films with multi-responsive actuation capability and high electromagnetic interference shielding performances. Carbon.

[63] Jiang C, Tan D, Li Q, Huang J, Bu J (2021). High-performance and reliable silver nanotube networks for efficient and large-scale transparent electromagnetic interference shielding. ACS Appl. Mater. Interfaces.

[64] Chung DDL (2000). Materials for electromagnetic interference shielding. J. Mater. Eng. Perform..

[65] Shu J-C, Cao W-Q, Cao M-S (2021). Diverse metal-organic framework architectures for electromagnetic absorbers and shielding. Adv. Funct. Mater..

[66] Pang K, Liu X, Liu Y, Chen Y, Xu Z (2021). Highly conductive graphene film with high-temperature stability for electromagnetic interference shielding. Carbon.

[67] Liang J, Wang Y, Huang Y, Ma Y, Liu Z (2009). Electromagnetic interference shielding of graphene/epoxy composites. Carbon.

[68] Li MX, Yang K, Zhu WG, Shen JH, Rollinson J (2020). Copper-coated reduced graphene oxide fiber mesh-polymer composite films for electromagnetic interference shielding. ACS Appl. Nano Mater..

[69] Liang C, Ruan K, Zhang Y, Gu J (2020). Multifunctional flexible electromagnetic interference shielding silver nanowires/cellulose films with excellent thermal management and Joule heating performances. ACS Appl. Mater. Interfaces.

[70] Liang C, Song P, Gu H, Ma C, Guo Y (2017). Nanopolydopamine coupled fluorescent nanozinc oxide reinforced epoxy nanocomposites. Compos. A Appl. S..

[71] Chung DDL (2019). A review of multifunctional polymer-matrix structural composites. Compos. B Eng..

[72] Umoren SA, Solomon MM (2019). Protective polymeric films for industrial substrates: a critical review on past and recent applications with conducting polymers and polymer composites/nanocomposites. Prog. Mater. Sci..

[73] Wang L, Qiu H, Liang C, Song P, Han Y (2019). Electromagnetic interference shielding MWCNT-Fe_3_O_4_@Ag/epoxy nanocomposites with satisfactory thermal conductivity and high thermal stability. Carbon.

[74] Wang L, Chen L, Song P, Liang C, Lu Y (2019). Fabrication on the annealed Ti_3_C_2_T_x_ MXene/Epoxy nanocomposites for electromagnetic interference shielding application. Compos. B Eng..

[75] He Q, Chen R, Li S, Wang Z, Wen F (2021). Excellent thermally conducting modified graphite nanoplatelets and MWCNTs/poly(phenylene sulfone) composites for high-performance electromagnetic interference shielding effectiveness. Compos. A Appl. S..

[76] Kwon S, Ma R, Kim U, Choi HR, Baik S (2014). Flexible electromagnetic interference shields made of silver flakes, carbon nanotubes and nitrile butadiene rubber. Carbon.

[77] Al-Saleh MH, Saadeh WH, Sundararaj U (2013). EMI shielding effectiveness of carbon based nanostructured polymeric materials: a comparative study. Carbon.

[78] Park Y, Baeg K-J, Kim C (2019). Solution-processed nonvolatile organic transistor memory based on semiconductor blends. ACS Appl. Mater. Interfaces.

[79] Leydecker T, Squillaci MA, Liscio F, Orgiu E, Samori P (2019). Controlling ambipolar transport and voltage inversion in solution-processed thin-film devices through polymer blending. Chem. Mater..

[80] Wang H, Zheng K, Zhang X, Ding X, Zhang Z (2016). 3D network porous polymeric composites with outstanding electromagnetic interference shielding. Compos. Sci. Technol..

[81] Das NC, Khastgir D, Chaki TK, Chakraborty A (2000). Electromagnetic interference shielding effectiveness of carbon black and carbon fibre filled EVA and NR based composites. Compos. A Appl. S..

[82] Duan H, Zhao M, Yang Y, Zhao G, Liu Y (2018). Flexible and conductive PP/EPDM/Ni coated glass fiber composite for efficient electromagnetic interference shielding. J. Mater. Sci. Mater. El..

[83] Liang C, Hu C, Zheng Y, Yan K, Zhu X (2018). Modification of isotactic polypropylene by silica nanocapsules via melt blending method. Polym. Compos..

[84] Deng S, Bai H, Liu Z, Zhang Q, Fu Q (2019). Toward supertough and heat-resistant stereocomplex-type polylactide/elastomer blends with impressive melt stability via in situ formation of graft copolymer during one-pot reactive melt blending. Macromolecules.

[85] Wang Y, Gu FQ, Ni LJ, Liang K, Marcus K (2017). Easily fabricated and lightweight PPy/PDA/AgNW composites for excellent electromagnetic interference shielding. Nanoscale.

[86] Saini P, Choudhary V, Singh BP, Mathur RB, Dhawan SK (2009). Polyaniline-MWCNT nanocomposites for microwave absorption and EMI shielding. Mater. Chem. Phys..

[87] Yun J, Kim H-I (2011). Electromagnetic interference shielding effects of polyaniline-coated multi-wall carbon nanotubes/maghemite nanocomposites. Polym. Bull..

[88] Loste J, Lopez-Cuesta JM, Billon L, Garay H, Save M (2019). Transparent polymer nanocomposites: an overview on their synthesis and advanced properties. Prog. Polym. Sci..

[89] Harito C, Bavykin DV, Yuliarto B, Dipojono HK, Walsh FC (2019). Polymer nanocomposites having a high filler content: synthesis, structures, properties, and applications. Nanoscale.

[90] Guo Y, Yang X, Ruan K, Kong J, Dong M (2019). Reduced graphene oxide heterostructured silver nanoparticles significantly enhanced thermal conductivities in hot-pressed electrospun polyimide nanocomposites. ACS Appl. Mater. Interfaces.

[91] Yan D-X, Ren P-G, Pang H, Fu Q, Yang M-B (2012). Efficient electromagnetic interference shielding of lightweight graphene/polystyrene composite. J. Mater. Chem..

[92] Jia LC, Yan DX, Jiang X, Pang H, Gao JF (2018). Synergistic effect of graphite and carbon nanotubes on improved electromagnetic interference shielding performance in segregated composites. Ind. Eng. Chem. Res..

[93] Lv Z, Jia CL, Ji X, Yan DX, Lei J (2018). Repeatable, room-temperature-processed baroplastic-carbon nanotube composites for electromagnetic interference shielding. J. Mater. Chem. C.

[94] Vovchenko L, Matzui L, Oliynyk V, Launets V, Mamunya Y (2018). Nanocarbon/polyethylene composites with segregated conductive network for electromagnetic interference shielding. Mol. Cryst. Liq. Cryst..

[95] Cui C-H, Yan D-X, Pang H, Jia L-C, Bao Y (2016). Towards efficient electromagnetic interference shielding performance for polyethylene composites by structuring segregated carbon black/graphite networks. Chin. J. Polym. Sci..

[96] Yan D-X, Pang H, Li B, Vajtai R, Xu L (2015). Structured reduced graphene oxide/polymer composites for ultra-efficient electromagnetic interference shielding. Adv. Funct. Mater..

[97] Sun RH, Zhang HB, Liu J, Xie X, Yang R (2017). Highly conductive transition metal carbide/carbonitride(MXene)@polystyrene nanocomposites fabricated by electrostatic assembly for highly efficient electromagnetic interference shielding. Adv. Funct. Mater..

[98] Zhan Y, Wang J, Zhang K, Li Y, Meng Y (2018). Fabrication of a flexible electromagnetic interference shielding Fe_3_O_4_@reduced graphene oxide/natural rubber composite with segregated network. Chem. Eng. J..

[99] Liang C, Song P, Qiu H, Huangfu Y, Lu Y (2019). Superior electromagnetic interference shielding performances of epoxy composites by introducing highly aligned reduced graphene oxide films. Compos. A Appl. S..

[100] Ma Z, Wei A, Li Y, Shao L, Zhang H (2021). Lightweight, flexible and highly sensitive segregated microcellular nanocomposite piezoresistive sensors for human motion detection. Compos. Sci. Technol..

[101] Zhang LY, Liu M, Roy S, Chu EK, See KY (2016). Phthalonitrile-based carbon foam with high specific mechanical strength and superior electromagnetic interference shielding performance. ACS Appl. Mater. Interfaces.

[102] Shen B, Li Y, Zhai WT, Zheng WG (2016). Compressible graphene-coated polymer foams with ultralow density for adjustable electromagnetic interference (EMI) shielding. ACS Appl. Mater. Interfaces.

[103] Fan ZM, Wang DL, Yuan Y, Wang YS, Cheng ZJ (2020). A lightweight and conductive MXene/graphene hybrid foam for superior electromagnetic interference shielding. Chem. Eng. J..

[104] Zhang H, Zhang G, Gao Q, Tang M, Ma Z (2020). Multifunctional microcellular PVDF/Ni-chains composite foams with enhanced electromagnetic interference shielding and superior thermal insulation performance. Chem. Eng. J..

[105] Eswaraiah V, Sankaranarayanan V, Ramaprabhu S (2011). Functionalized graphene-PVDF foam composites for EMI shielding. Macromol. Mater. Eng..

[106] Zhang HM, Zhang GC, Tang M, Zhou LS, Li JT (2018). Synergistic effect of carbon nanotube and graphene nanoplates on the mechanical, electrical and electromagnetic interference shielding properties of polymer composites and polymer composite foams. Chem. Eng. J..

[107] Fan X, Zhang G, Gao Q, Li J, Shang Z (2019). Highly expansive, thermally insulating epoxy/Ag nanosheet composite foam for electromagnetic interference shielding. Chem. Eng. J..

[108] Wang L, Qiu H, Song P, Zhang Y, Lu Y (2019). 3D Ti_3_C_2_T_x_ MXene/C hybrid foam/epoxy nanocomposites with superior electromagnetic interference shielding performances and robust mechanical properties. Compos. A Appl. S..

[109] Zeng Z, Jin H, Chen M, Li W, Zhou L (2016). Lightweight and anisotropic porous MWCNT/WPU composites for ultrahigh performance electromagnetic interference shielding. Adv. Funct. Mater..

[110] Zeng ZH, Wang CX, Zhang YF, Wang PY, Shahabadi SIS (2018). Ultralight and highly elastic graphene/lignin-derived carbon nanocomposite aerogels with ultrahigh electromagnetic interference shielding performance. ACS Appl. Mater. Interfaces.

[111] Zeng ZH, Wu TT, Han DX, Ren Q, Siqueira G (2020). Ultralight, flexible, and biomimetic nanocellulose/silver nanowire aerogels for electromagnetic interference shielding. ACS Nano.

[112] Zeng ZH, Wang CX, Siqueira G, Han DX, Huch A (2020). Nanocellulose-MXene biomimetic aerogels with orientation-tunable electromagnetic interference shielding performance. Adv. Sci..

[113] Zhou Z-H, Li M-Z, Huang H-D, Li L, Yang B (2020). Structuring hierarchically porous architecture in biomass-derived carbon aerogels for simultaneously achieving high electromagnetic interference shielding effectiveness and high absorption coefficient. ACS Appl. Mater. Interfaces.

[114] Liang C, Song P, Ma A, Shi X, Gu H (2019). Highly oriented three-dimensional structures of Fe_3_O_4_ decorated CNTs/reduced graphene oxide foam/epoxy nanocomposites against electromagnetic pollution. Compos. Sci. Technol..

[115] Wang QW, Zhang HB, Liu J, Zhao S, Xie X (2019). Multifunctional and water-resistant MXene-Decorated polyester textiles with outstanding electromagnetic interference shielding and Joule heating performances. Adv. Funct. Mater..

[116] Lin S, Liu J, Wang Q, Zu D, Wang H (2019). Highly robust, flexible, and large-scale 3D-Metallized sponge for high-performance electromagnetic interference shielding. Adv. Mater. Technol..

[117] Van-Tam N, Min BK, Yi Y, Kim SJ, Choi C-G (2020). MXene(Ti_3_C_2_T_x_)/graphene/PDMS composites for multifunctional broadband electromagnetic interference shielding skins. Chem. Eng. J..

[118] Liang C, Liu Y, Ruan Y, Qiu H, Song P (2020). Multifunctional sponges with flexible motion sensing and outstanding thermal insulation for superior electromagnetic interference shielding. Compos. A Appl. S..

[119] Zhou T, Xu C, Liu H, Wei Q, Wang H (2020). Second time-scale synthesis of high-quality graphite films by quenching for effective electromagnetic interference shielding. ACS Nano.

[120] Zhang X, Wang X, Lei Z, Wang L, Tian M (2020). Flexible MXene-Decorated fabric with interwoven conductive networks for integrated Joule heating, electromagnetic interference shielding, and strain sensing performances. ACS Appl. Mater. Interfaces.

[121] Liu J, Liu ZS, Zhang HB, Chen W, Zhao ZF (2020). Ultrastrong and highly conductive MXene-Based films for high-performance electromagnetic interference shielding. Adv. Electron. Mater..

[122] Luo JQ, Zhao S, Zhang HB, Deng ZM, Li LL (2019). Flexible, stretchable and electrically conductive MXene/natural rubber nanocomposite films for efficient electromagnetic interference shielding. Compos. Sci. Technol..

[123] Ma Z, Kang S, Ma J, Shao L, Wei A (2019). High-Performance and rapid-response electrical heaters based on ultraflexible, heat-resistant, and mechanically strong aramid nanofiber/Ag nanowire nnanocomposite papers. ACS Nano.

[124] Ma Z, Kang S, Ma J, Shao L, Zhang Y (2020). Ultraflexible and mechanically strong double-layered aramid nanofiber-Ti_3_C_2_T_x_ MXene/silver nanowire nanocomposite papers for high-performance electromagnetic interference shielding. ACS Nano.

[125] Zhou B, Zhang Z, Li Y, Han G, Feng Y (2020). Flexible, robust, and multifunctional electromagnetic interference shielding film with alternating cellulose nanofiber and MXene layers. ACS Appl. Mater. Interfaces.

[126] Zhang YL, Wang L, Zhang JL, Song P, Xiao ZR (2019). Fabrication and investigation on the ultra-thin and flexible Ti_3_C_2_T_x_/co-doped polyaniline electromagnetic interference shielding composite films. Compos. Sci. Technol..

[127] Zhan YH, Lago E, Santillo C, Castillo AED, Hao S (2020). An anisotropic layer-by-layer carbon nanotube/boron nitride/rubber composite and its application in electromagnetic shielding. Nanoscale.

[128] Yang W, Liu J-J, Wang L-L, Wang W, Yuen ACY (2020). Multifunctional MXene/natural rubber composite flms with exceptional flexibility and durability. Compos. B Eng..

[129] Jin X, Wang J, Dai L, Liu X, Li L (2020). Flame-retardant poly(vinyl alcohol)/MXene multilayered films with outstanding electromagnetic interference shielding and thermal conductive performances. Chem. Eng. J..

[130] Ren W, Zhu H, Yang Y, Chen Y, Duan H (2020). Flexible and robust silver coated non-woven fabric reinforced waterborne polyurethane flms for ultra-effcient electromagnetic shielding. Compos. B Eng..

[131] Xu YD, Yang YQ, Yan DX, Duan HJ, Zhao GZ (2018). Gradient structure design of flexible waterborne polyurethane conductive films for ultraefficient electromagnetic shielding with low reflection characteristic. ACS Appl. Mater. Interfaces.

[132] Zhang J, Kong N, Uzun S, Levitt A, Seyedin S (2020). Scalable manufacturing of free-standing, strong Ti_3_C_2_T_x_ MXene films with outstanding conductivity. Adv. Mater..

[133] Jia L-C, Zhou C-G, Sun W-J, Xu L, Yan D-X (2020). Water-based conductive ink for highly efficient electromagnetic interference shielding coating. Chem. Eng. J..

[134] Xi JB, Li YL, Zhou EZ, Liu YJ, Gao WW (2018). Graphene aerogel films with expansion enhancement effect of high-performance electromagnetic interference shielding. Carbon.

[135] Yuan C, Huang J, Dong Y, Huang X, Lu Y (2020). Record-High transparent electromagnetic interference shielding achieved by simultaneous microwave fabry-perot interference and optical antireflection. ACS Appl. Mater. Interfaces.

[136] Chen L, Zhao J, Wang L, Peng F, Liu H (2019). In-situ pyrolyzed polymethylsilsesquioxane multi-walled carbon nanotubes derived ceramic nanocomposites for electromagnetic wave absorption. Ceram. Int..

[137] Wang HY, Ji CG, Zhang C, Zhang YL, Zhang Z (2019). Highly transparent and broadband electromagnetic interference shielding based on ultrathin doped Ag and conducting oxides hybrid film structures. ACS Appl. Mater. Interfaces.

[138] Gu J, Hu S, Ji H, Feng H, Zhao W (2020). Multi-layer silver nanowire/polyethylene terephthalate mesh structure for highly efficient transparent electromagnetic interference shielding. Nanotechnology.

[139] Zhu X, Xu J, Qin F, Yan Z, Guo A (2020). Highly efficient and stable transparent electromagnetic interference shielding films based on silver nanowires. Nanoscale.

[140] Chen W, Liu L-X, Zhang H-B, Yu Z-Z (2020). Flexible, transparent, and conductive Ti_3_C_2_T_x_ MXene-Silver nanowire films with smart acoustic sensitivity for high-performance electromagnetic interference shielding. ACS Nano.

[141] Zhan Y, Hao X, Wang L, Jiang X, Cheng Y (2021). Superhydrophobic and flexible silver nanowire-coated cellulose filter papers with sputter-deposited nickel nanoparticles for ultrahigh electromagnetic interference shielding. ACS Appl. Mater. Interfaces.

[142] Li X-H, Li X, Liao K-N, Min P, Liu T (2016). Thermally annealed anisotropic graphene aerogels and their electrically conductive epoxy composites with excellent electromagnetic interference shielding efficiencies. ACS Appl. Mater. Interfaces.

[143] Chen Y, Zhang H-B, Huang Y, Jiang Y, Zheng W-G (2015). Magnetic and electrically conductive epoxy/graphene/carbonyl iron nanocomposites for efficient electromagnetic interference shielding. Compos. Sci. Technol..

[144] Liang C, Song P, Qiu H, Zhang Y, Ma X (2019). Constructing interconnected spherical hollow conductive networks in silver platelets/reduced graphene oxide foam/epoxy nanocomposites for superior electromagnetic interference shielding effectiveness. Nanoscale.

[145] Liang C, Qiu H, Han Y, Gu H, Song P (2019). Superior electromagnetic interference shielding 3D graphene nanoplatelets/reduced graphene oxide foam/epoxy nanocomposites with high thermal conductivity. J. Mater. Chem. C.

[146] Gao W, Zhao N, Yu T, Xi J, Mao A (2020). High-efficiency electromagnetic interference shielding realized in nacre-mimetic graphene/polymer composite with extremely low graphene loading. Carbon.

[147] Fang H, Guo H, Hu Y, Ren Y, Hsu P-C (2020). In-situ grown hollow Fe_3_O_4_ onto graphene foam nanocomposites with high EMI shielding effectiveness and thermal conductivity. Compos. Sci. Technol..

[148] Shen ZM, Feng JC (2019). Preparation of thermally conductive polymer composites with good electromagnetic interference shielding efficiency based on natural wood-derived carbon scaffolds. ACS Sustain. Chem. Eng..

[149] Huangfu Y, Liang C, Han Y, Qiu H, Song P (2019). Fabrication and investigation on the Fe_3_O_4_/thermally annealed graphene aerogel/epoxy electromagnetic interference shielding nanocomposites. Compos. Sci. Technol..

[150] Wang L, Song P, Lin C-T, Kong J, Gu J (2020). 3D shapeable, superior electrically conductive cellulose nanofibers/Ti_3_C_2_T_x_ MXene aerogels/epoxy nanocomposites for promising EMI shielding. Research.

[151] Huangfu Y, Ruan K, Qiu H, Lu Y, Liang C (2019). Fabrication and investigation on the PANI/MWCNT/thermally annealed graphene aerogel/epoxy electromagnetic interference shielding nanocomposites. Compos. A Appl. S..

[152] Song P, Liang C, Wang L, Qiu H, Gu H (2019). Obviously improved electromagnetic interference shielding performances for epoxy composites via constructing honeycomb structural reduced graphene oxide. Compos. Sci. Technol..

